# Building predictive in vitro pulmonary toxicity assays using high-throughput imaging and artificial intelligence

**DOI:** 10.1007/s00204-018-2213-0

**Published:** 2018-04-28

**Authors:** Jia-Ying Joey Lee, James Alastair Miller, Sreetama Basu, Ting-Zhen Vanessa Kee, Lit-Hsin Loo

**Affiliations:** Bioinformatics Institute, Agency for Science, Technology, and Research, 30 Biopolis Street, #07-01 Matrix, Singapore, 138671 Singapore

## Abstract

Human lungs are susceptible to the toxicity induced by soluble xenobiotics. However, the direct cellular effects of many pulmonotoxic chemicals are not always clear, and thus, a general in vitro assay for testing pulmonotoxicity applicable to a wide variety of chemicals is not currently available. Here, we report a study that uses high-throughput imaging and artificial intelligence to build an in vitro pulmonotoxicity assay by automatically comparing and selecting human lung-cell lines and their associated quantitative phenotypic features most predictive of in vivo pulmonotoxicity. This approach is called “High-throughput In vitro Phenotypic Profiling for Toxicity Prediction” (HIPPTox). We found that the resulting assay based on two phenotypic features of a human bronchial epithelial cell line, BEAS-2B, can accurately classify 33 reference chemicals with human pulmonotoxicity information (88.8% balance accuracy, 84.6% sensitivity, and 93.0% specificity). In comparison, the predictivity of a standard cell-viability assay on the same set of chemicals is much lower (77.1% balanced accuracy, 84.6% sensitivity, and 69.5% specificity). We also used the assay to evaluate 17 additional test chemicals with unknown/unclear human pulmonotoxicity, and experimentally confirmed that many of the pulmonotoxic reference and predicted-positive test chemicals induce DNA strand breaks and/or activation of the DNA-damage response (DDR) pathway. Therefore, HIPPTox helps us to uncover these common modes-of-action of pulmonotoxic chemicals. HIPPTox may also be applied to other cell types or models, and accelerate the development of predictive in vitro assays for other cell-type- or organ-specific toxicities.

**Electronic supplementary material:**

The online version of this article (10.1007/s00204-018-2213-0) contains supplementary material, which is available to authorized users.

## Introduction

Human lungs are exposed to inhaled or blood-borne soluble xenobiotics that may originate from the environment, food, consumer products, and/or pharmaceuticals. In the lungs, bronchial and alveolar epithelial cells (BECs and AECs) are major sites of xenobiotic metabolism, and thus susceptible to the toxicity induced by these foreign chemicals (Devereux et al. 1993; Foth 1995; Courcot et al. 2012). For example, bleomycin, methotrexate, and temsirolimus (three intravenously or orally delivered anti-cancer drugs) may cause pulmonary fibrosis, pneumonitis, and/or other lung diseases (Blum et al. 1973; Lateef et al. 2005; Duran et al. 2006). Excessive exposures to diacetyl (a food and beverage flavoring chemical) or paraquat (an agricultural chemical) may also lead to bronchiolitis obliterans (Kreiss et al. 2002) or pulmonary edema (Dinis-Oliveira et al. 2008), respectively. Despite the known adverse pulmonary effects of these xenobiotics in humans, the key cellular effects, or “modes-of-action” (MoA) (Seed et al. 2005), of these chemicals in human lung cells are not always clear. Do these known pulmonotoxic chemicals, which may have diverse chemical structures and intracellular targets, induce similar or different MoAs in the lung cells? Are in vitro cell-viability or death endpoints indicative or even predictive of the in vivo pulmonotoxicity of these chemicals? The answers to these questions are critical for the development of predictive in vitro pulmonotoxicity assays.

The need of predictive alternative assays is especially pertinent to pulmonary toxicity. A survey of 142 drugs approved between 2001 and 2010 found that only 19% of the pulmonary adverse drug reactions identified post-marketing could have been predicted based on pre-clinical animal studies (Tamaki et al. 2013). For example, pre-clinical assessments of temsirolimus, carbamazepine, and tenofovir did not find any major adverse pulmonary effect in rodents (Ciba-Geigy Corp 1967; Gilead Sciences 2001; Wyeh Pharmaceuticals 2007), but these drugs were later found to cause interstitial lung disease, pneumonitis, or pneumonia in humans (Wilschut et al. 1997; Gilead Sciences 2001; Duran et al. 2006). On the other hand, there are chemicals, such as butylated hydroxytoluene (BHT, an antioxidant and food additive), that may induce pulmonary edema or other lesions in animals but not in humans (Witschi et al. 1993). Furthermore, even closely related species may have discrepancies in their pulmonary responses. A survey found that there is no concordance between mouse and rat non-carcinogenic lung lesions observed in acute and long-term rodent studies of 37 chemicals (Wang and Gray 2015). All of these findings highlight the limitations of animal models in predicting human pulmonary toxicity, and the urgent need for developing more predictive alternative assays.

**Fig. 1:**
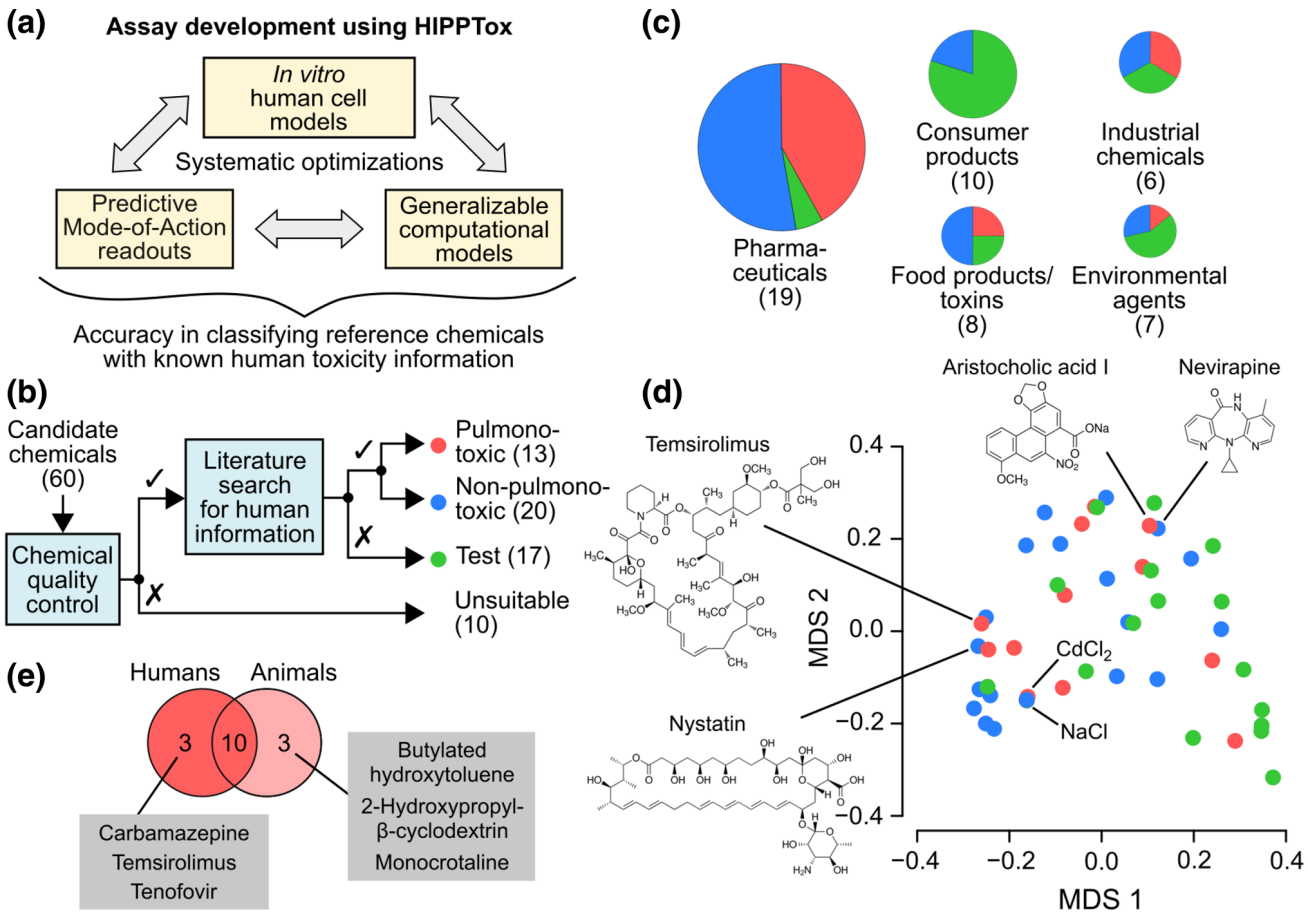
Reference chemicals with known human toxicity information are required to build a predictive in vitro toxicity assay. **a** High-throughput imaging and artificial intelligence can be used to systematically and efficiently optimize the three inter-dependent components of an in vitro toxicity assay and achieve a higher overall prediction accuracy. We call this approach “High-throughput In-vitro Phenotypic Profiling for Toxicity Prediction” (HIPPTox). **b** Schematic showing the reference and test chemical selection process starting from a list of 60 candidate chemicals. **c** Categorization of the reference and test chemicals according to their sources or applications. **d** Multi-dimensional scaling plot showing the chemical structure dissimilarities based on Tanimoto coefficients between all the reference and test compounds (MDS1/2 = the first and second coordinates of the multi-dimensional scaling). **e** Venn diagram showing the overlap between reference chemicals that are known to be directly pulmonotoxic to humans or animals (mostly rodents). (Red = pulmonotoxic reference chemicals, blue = non-pulmonotoxic reference chemicals, green = test chemicals.) (Color figure online)

The construction of a predictive assay for cell-type-specific toxicity requires systematic optimizations of three inter-dependent components (Fig. 1a): (1) an in vitro human cell model that can mimic, to a certain extent, in vivo human cell-type-specific responses to xenobiotics; (2) quantitative in vitro phenotypic readouts based on the cell model that can reflect the MoAs of xenobiotics toxic to the cell type; and (3) computational models or classifiers based on the readouts that can optimally distinguish between the effects of xenobiotics that are toxic or non-toxic to the cell type. The development of such an assay often requires balancing between the performances, requirements, and costs of these three individual components (Fig. 1a). For example, advanced in vitro human lung-cell models, such as 3D airway epithelial tissue (Kelly BéruBé et al. 2009; Sauer et al. 2013) or microfluidic-chip-based (Huh et al. 2010) models, may better mimic the in vivo physiology of lung cells or tissues, but they often require complicated, expensive, and lower throughput experimental procedures or devices. In addition, their added values over 2D immortalized lung-cell lines have not been clearly demonstrated in large-scale studies (Sauer et al. 2013). Furthermore, due to the biological differences between different in vitro models, the same chemicals may induce different molecular or phenotypic changes in these models. Therefore, optimum in vitro toxicity readouts (or “markers”) needs to be determined specifically for each in vitro model. This would require the evaluation of different potential phenotypic readouts using large numbers of reference chemicals with known human pulmonotoxicity information (Fig. 1a). However, most existing approaches simply use standard cell-death or viability endpoints, such as lactate dehydrogenase (LDH) leakage, tetrazolium salt (MTT) conversion, or propidium iodide (PI) stains, as in vitro pulmonotoxicity markers (Bargout et al. 2000; Sauer et al. 2013; Hess et al. 2016). These and other similar endpoints have been repeatedly shown in large-scale studies to be poorly predictive of cell-type-specific toxicities (Lin and Will 2012; Sison-Young et al. 2017). Finally, to construct generalizable computational models, evaluations of different modeling techniques using large numbers of reference chemicals are again required. However, the availability of human data is scarce, especially for reference chemicals that are known to be non-pulmonotoxic. Thus, systematic evaluations and optimizations of these three components have rarely been performed. Due to all of these problems, the development of a predictive in vitro pulmonotoxicity assay remains very challenging.

To build a predictive in vitro pulmonotoxicity assay, our strategy is to use high-throughput imaging and artificial intelligence to systematically and efficiently optimize the three components of the assay (Fig. 1a). This approach is called “High-Throughput In vitro Phenotypic Profiling for Toxicity Prediction” (HIPPTox), and consists of two main steps (Bougen-Zhukov et al. 2017; Loo and Zink 2017). In the first step, we automatically measure large numbers of cellular phenotypic features based on microscopy images of human cells that have been exposed to a set of reference chemicals. For example, these features may include all possible pairwise ratios between the total intensity values of different stained fluorescent markers at different subcellular regions. Unlike conventional high-content analysis, HIPPTox does not start with pre-assumed toxicity MoAs or specific phenotypic readouts designed to reflect these MoAs (Bougen-Zhukov et al. 2017; Loo and Zink 2017). In the second step, we use machine learning methods to automatically search for the most predictive phenotypic readouts from all the measured features. Thus, HIPPTox may discover novel or unexpected MoAs for the reference chemicals. This would also allow us to compare the performances of different in vitro cell models. We have previously used HIPPTox to build a highly predictive in vitro nephrotoxicity assay by systematically comparing the phenotypic features of primary and immortalized human proximal tubule cells (Su et al. 2016; Loo and Zink 2017).

Here, we report a subsequent study to use HIPPTox to build an in vitro pulmonotoxicity assay based on 33 reference chemicals with published human pulmonotoxicity information. For in vitro human cell models, we evaluated two different human lung-cell lines, namely, BEAS-2B (a BEC line) and A549 (an AEC line). These cell lines do not have the problem of inter-individual variability commonly observed in primary human lung cells (Cohen et al. 1979; Courcot et al. 2012), and can be easily stored and expanded for future large-scale experiments. For in vitro pulmonotoxicity markers, we evaluated 165 candidate phenotypic features extracted from microscopy images of these cell lines. For the computational model, we developed a cascade classifier that used a small subset of the extracted features in succession to predict pulmonotoxicity. The result is an optimized in vitro assay based on two phenotypic features of the BEAS-2B cell line that can achieve 88.8% balance accuracy, 84.6% sensitivity, and 93.0% specificity. These two features are changes in cell count and spatial cross correlation between DNA and phosphorylated histone H2AX (γH2AX). We also applied the pulmonotoxicity assay to test 17 additional chemicals of interest with unknown/unclear human pulmonotoxicity, and experimentally confirmed that most of the pulmonotoxic reference and predicted-positive test chemicals induced DNA strand breaks and/or activation of the DNA-damage response (DDR) pathway. Therefore, our results show that DDR is a common toxicity mode-of-action represented by these two features, and induced by many pulmonotoxic chemicals with diverse chemical structures. Our assay may be used to efficiently predict the potential pulmonotoxicity of these chemicals.

## Results

### Compilation of a reference chemical list

To use HIPPTox, a list of reference chemicals that are known to be directly toxic or non-toxic to the human lungs is required (Fig. 1a). We started with 60 chemicals of interest, many of which are well-established human pulmonotoxicants, such as bleomycin, amiodarone, and paraquat (Blum et al. 1973; Martin and Rosenow 1988; Dinis-Oliveira et al. 2008). We also included chemicals with high human exposure levels, such as phthalates and naphthalenes, whose pulmonary safety are unclear but of major concerns and interests. However, we found that ten of the candidate chemicals are either poorly soluble, highly autofluorescent (Supplementary Material 1—Fig. S1), or producing imaging artifacts, and thus not suitable for our imaging assays (Fig. 1b and Supplementary Material 1—Table S1). For example, benzo[α]pyrene (a commonly studied lung toxicant that has five aromatic rings) was found to have strong autofluorescence in all three fluorescence channels that we used for imaging (Supplementary Material 1—Fig. S2 and Table S1). Our results show that, to avoid false detection of chemical effects, autofluorescence of all chemicals must be checked before performing large-scale fluorescence-based assays. The remaining 50 chemicals consist of pharmaceuticals, consumer product ingredients, food ingredients or toxins, industrial chemicals, or environmental agents (Fig. 1c). They have diverse chemical structures, ranging from simple molecules, such as cadmium chloride and sodium chloride, to complex molecules, such as temsirolimus and nystatin (Fig. 1d). Before our study, it was unclear whether these diverse chemicals may directly injure lung cells, and whether they induce similar or different MoAs in the lung cells.

In our study, we define “pulmonotoxicity” as the potential hazard to directly cause pulmonary edema, fibrosis, pneumonitis, necrosis, and/or other damages to the lungs in humans. The availability of in vivo human pulmonary toxicity data for most chemicals is scarce. Among the 50 chemicals, we could find reliable and relevant human data for 33 of them based on published expert reviews/reports, clinical studies, post-marketing safety surveillance, poisoning-incident reports, and/or epidemiological studies (“Materials and methods”, Fig. 1b and Supplementary Material 1—Table S2). We did not use any information from in vitro human cell lines. Based on the compiled in vivo human data, we assigned 13 of these chemicals to the “pulmonotoxic” class and 20 to the “non-pulmonotoxic” class (Fig. 1b). Together, these two classes of chemicals were also called the “reference” chemicals. The rest of the chemicals had unknown or unclear human data, and thus were assigned to the “test” class. These chemicals were not used to train or evaluate our models or phenotypic readouts, but their pulmonotoxicity was predicted using the final assay based on the reference chemicals.

To illustrate the process, the annotations for a few selected chemicals are briefly described here. Occupational exposure to diacetyl was found to be associated with bronchiolitis obliterans (Kreiss et al. 2002; van Rooy et al. 2007), and thus the chemical was annotated as “pulmonotoxic”. Ketoconazole (an antifungal drug) and nevirapine (a human-immunodeficiency-virus drug) may induce acute liver injury (Rodríguez et al. 1999; Patel et al. 2004), but no major pulmonotoxicity has been reported in patients who took the drugs (Sugar et al. 1987; Patel et al. 2004). Similarly, monocrotaline, a plant-derived pyrrolizidine alkaloid, may cause pulmonary hypertension and edema in rats and other animals; but its main targets are liver and endothelial cells, and its pulmonary effects are indirectly due to metabolites released from the liver (Huxtable 1993). Furthermore, “there is no evidence of involvement of organs other than the liver and central nervous system ascribed primarily to pyrrolizidine alkaloid toxicity in any of the published human case reports” (WHO-IPCS 1988). Therefore, all of these chemicals were annotated as “non-pulmonotoxic” in our study. Overdose of *p*-phenylenediamine (a hair-dye ingredient) leads to pulmonary obstruction and edema, but the effects may be indirectly due to severe muscle damage or “rhabdomyolysis” that can damage lung cells (Abidi et al. 2008; Behera et al. 2015). Therefore, the direct effect of this chemical to lung cells was unclear, and it was annotated as a “test” chemical. Finally, we could not find any relevant human pulmonary information for dibutyl and diethyl phthalates, and naphthalene, and 1-nitronaphthalene. Thus, they were also annotated as “test” chemicals. These reference chemicals allow us to systematically evaluate our models using HIPPTox. They may also be used as standard reference chemicals for other further studies or developments of more advanced alternative assays for pulmonotoxicity.

We also compiled relevant animal information (mostly from rodents) for all the 50 chemicals (Supplementary Material 1—Table S2). This information was only used for the purpose of comparison, and not used to derive the pulmonotoxicity annotations. We found that ten of the reference chemicals are known to be pulmonotoxic to both humans and animals, and six of the reference chemicals have different pulmonary effects in humans vs. animals (Fig. 1e). Specifically, pre-clinical assessments of carbamazepine, temsirolimus, and tenofovir (three pulmonotoxic drugs) found no major pulmonary effect in animals (Supplementary Material 1—Table S2). On the other hand, 2-hydroxypropyl-β-cyclodextrin, BHT, and monocrotaline were found to induce adverse pulmonary effects in animals, but similar effects have not been observed in humans (Supplementary Material 1—Table S2). The discrepancies highlight the importance of developing toxicity assays based on human and not animal information.

### High-throughput imaging of BEAS-2B and A549 cells

We compared two human lung-cell lines, namely, BEAS-2B (a BEC line) and A549 (an AEC line). A previous study of ten different human lung-cell lines, including BEAS-2B and A549, found that BEAS-2B has the highest correlation in the expressions of 380 genes involved in xenobiotic metabolism and disposition, and the lowest number of dysregulated genes, compared to human non-tumoral pulmonary parenchyma and bronchial mucosa tissues (Courcot et al. 2012). Thus, BEAS-2B may be more active in xenobiotic metabolism than A549. However, we still included A549, because it is a commonly used in vitro cell model for pulmonotoxicity (Kelly BéruBé et al. 2009).

**Fig. 2:**
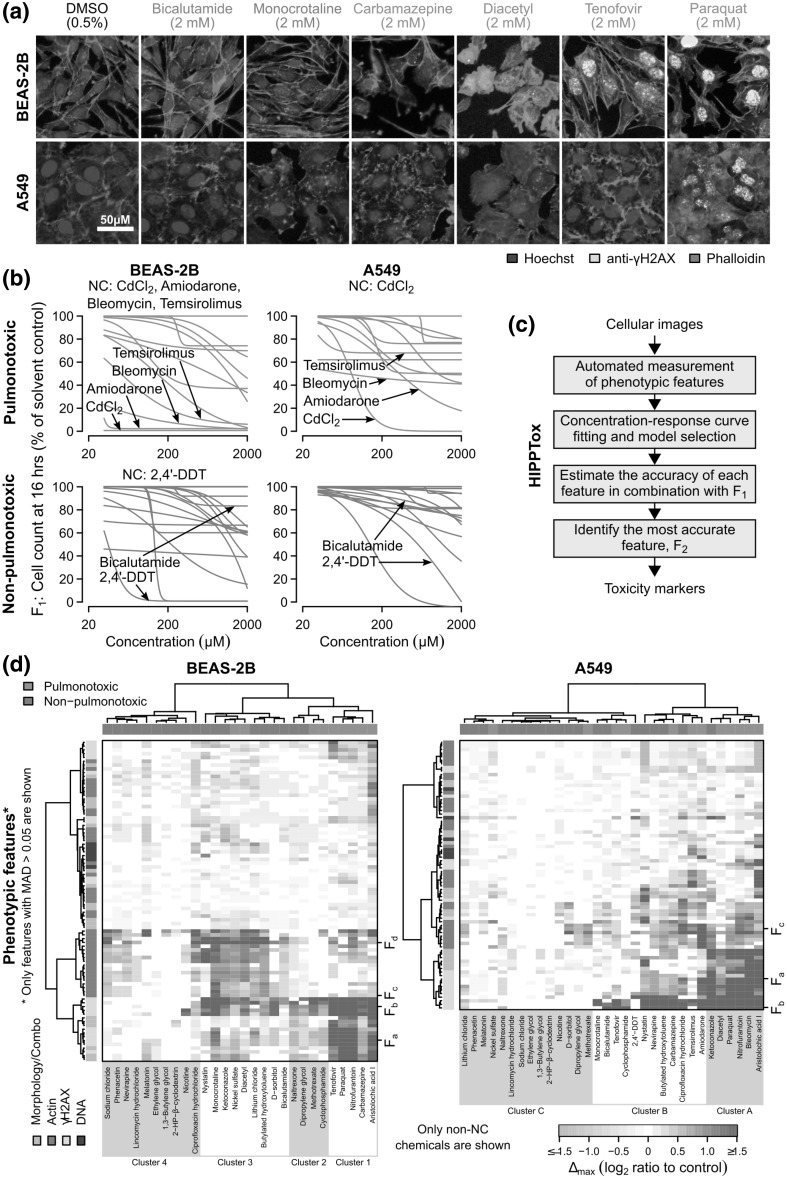
Image-based phenotypic profiling shows that BEAS-2B is more active than A549 cells. **a** Fluorescence microscopy images showing BEAS-2B and A549 cells that had been treated with six of the reference chemicals for 16 h (scale bar = 50 µm). **b** Percentages of cell count with respect to the solvent controls for all the 30 reference chemicals (red = pulmonotoxic, blue = non-pulmonotoxic reference chemicals). The values were quantified from the cellular images. For BEAS-2B cells, five chemicals, namely, 2,4′-DDT, amiodarone, bleomycin, cadmium(II) chloride (CdCl_2_), and temsirolimus were found to cause the “no cell” (NC) condition, which is defined to be < 30% cell count at ≥ 125 µM. For A549 cells, only CdCl_2_ was found to cause NC. **c** Schematic showing the key steps of HIPPTox used to automatically identify predictive toxicity markers from the obtained cellular images. The same procedures were repeated for both BEAS-2B and A549 cells. **d** Heatmaps showing changes in the measured phenotypic features of BEAS-2B and A549 cells (“∆_max_”, see main text) induced by the 33 reference chemicals. Only features with median absolute deviation (MAD) > 0.05 are shown (F_a_ = mean nuclear γH2AX intensity, F_b_ = number of γH2AX foci, F_c_ = total cellular actin intensity, F_d_ = mean actin object size). The vertical or horizontal groupings of the feature values were based on hierarchical clustering of the columns (chemicals) or rows (features) of the heatmaps, respectively. The Ward’s method (“ward.D” in the hclust() function) was used to agglomerate the clusters. (Color figure online)

After treating the two cell lines with the 50 chemicals in seven concentrations for 16 h, we stained the cells with four fluorescent markers, and imaged them using a high-throughput imaging system (“Materials and methods”, Fig. 2a, and Supplementary Material 1—Fig. S3). In theory, HIPPTox may be used to screen a large set of fluorescent markers that reflect different biological or cellular processes. However, such a screen was not performed, because we could already find phenotypic features with the desired prediction accuracy based on these four fluorescent markers. Specifically, Hoechst and CellMask were used to stain the nucleus and whole cell, and may reflect early and late phenotypes of apoptotic cells, such as condensation or fragmentation of chromosomes, and blebbing or rupture of plasma membranes. Phalloidin was used to stain the actin filaments, because cytoskeleton remodeling is a key event during the repair of lung epithelial cell injury (Crosby and Waters 2010). Finally, an antibody that specifically stains phosphorylated histone H2AX (γH2AX) was used to measure DDR (Rogakou et al. 1998). The marker was motivated by results from two recent studies. The first study found that DDR is commonly induced by diverse xenobiotics in human proximal tubule cells, although many of these chemicals are not known to target DNA directly (Su et al. 2016). The second study found that house-dust mite (a lung allergen) can cause DDR in human lung epithelial cells under both in vivo and in vitro conditions (Chan et al. 2016). Therefore, we hypothesized that, similar to the proximal tubule cells, DDR may also be used as a cellular-stress marker in the lung cells, and induced by pulmonotoxic chemicals that do not necessarily directly interact with or damage DNA. Importantly, we did not manually design phenotypic readouts for any specific MoA, and still used HIPPTox to automatically measure and screen large numbers of features based on these fluorescent markers.

### BEAS-2B cells is more sensitive to the reference chemicals

After a relatively short exposure of 16 h, how many of the reference chemicals can kill BEAS-2B or A549 cells? For most of the tested concentrations, we identified up to ~ 2500 cells from the acquired images (Fig. 2a and Supplementary Material 1—Fig. S3) using automated image processing algorithms (Laksameethanasan et al. 2013). The number of cells identified from the images (“*F*_1_”) can be used as a proxy indicator of cell death/health, because dead or injured cells would detach from the imaging plates. In BEAS-2B cells, we found that 4 of the 13 pulmonotoxic chemicals, namely, amiodarone, bleomycin, cadmium(II) chloride, and temsirolimus, caused < 30% cell count at concentrations ≥ 125 µM (Fig. 2b). In this report, we denote this condition as “no cell” (or “NC”). Among those four NC-causing chemicals, cadmium(II) chloride, and amiodarone also caused close to 100% cell lost at most of the tested concentrations (Fig. 2b). Surprisingly, in A549 cells, we only found one NC-causing chemical, namely, cadmium(II) chloride (Fig. 2b). We also found that most of the non-pulmonotoxic chemicals (17/20) caused more or similar levels of cell lost in BEAS-2B cells than A549 cells at the highest tested concentration (Fig. 2b). All of these results suggest that BEAS-2B is more sensitive to the reference chemicals than A549 cells, irrespective of whether the chemicals are pulmonotoxic or not. This is consistent to the previous finding that BEAS-2B may be more metabolically active than A549 cells (Courcot et al. 2012).

However, the noticeable cell loss at 16 h induced by non-pulmonotoxic chemicals in both BEAS-2B and A549 cells (Fig. 2b) also suggests that cell count (or other related cell health/death endpoints) at this timepoint may not be sufficient to predict pulmonotoxicity. We hypothesized that, beyond cell death, pulmonotoxic chemicals may induce other changes in cellular phenotypes that are more predictive of pulmonotoxicity. In a later section, we will present the results on cell-viability measurement based on a standard resazurin assay after 72 h of chemical exposure, and conclusively compare the predictivity of cell-viability and phenotypic features.

Among all the reference chemicals, we found an obvious outlier, 2,4′-DDT (a constituent of an organochloride insecticide), which was annotated as “non-pulmonotoxic” based on published reports (Supplementary Material 1—Table S2). The chemical consistently showed high levels of cell loss in both cell lines, especially in BEAS-2B cells (Fig. 2b). The discrepancy between the in vivo and in vitro effects of 2,4′-DDT may be due to its rapid metabolism and excretion in the human body, as shown in previous in vivo human studies (Morgan and Roan 1974). BEAS-2B cells may have much higher accumulation of 2,4′-DDT than the lungs in vivo, and thus may be more sensitive to the chemical. Another possibility is that DDT and its metabolites (such as dichlorodiphenyldichloroethylene or DDE) may have different affinities to their targets. Therefore, a difference in the in vitro and in vivo metabolism rates of 2,4′-DDT may lead to different downstream effects.

### Phenotypic profiling of BEAS-2B and A549 cells

During the first step of HIPPTox (Fig. 2c), we automatically measured 165 phenotypic features from every single cell stained with the four fluorescent markers (Supplementary Material 1—Table S3). The features include 65 texture features (measuring the statistics of the spatial co-occurrence patterns of the markers), 36 intensity features (measuring the staining levels of the markers at different subcellular regions), 29 intensity ratio features (measuring the ratios between intensity features), 18 correlation features (measuring the spatial correlations between two markers at the single-cell level), and 17 morphology features (measuring the shape properties of the nuclear and cellular regions). We also included cell count as a feature. Compared to our previous work (Su et al. 2016), we have added two new functions to HIPPTox for detecting binary objects (i.e., neighboring pixels with similar and relatively high intensity levels of the stained fluorescent markers) and “chromosomal” regions (i.e., sub-nuclear regions with more intense DNA staining levels) (Supplementary Material 1—Fig. S4). These functions allow us to detect, for example, the number of γH2AX objects/foci or the average intensity of γH2AX at the chromosomal region. Some of these phenotypic features were previously used to build assays for predicting nephrotoxicity (Su et al. 2016), cellular sensitivity to cytotoxic agents (Loo et al. 2017), drug targets/mechanisms (Loo et al. 2007), or protein functions (Loo et al. 2014). Thus, our current phenotypic feature set may also be discriminative enough to predict pulmonotoxicity.

For each phenotypic feature, we first computed the log_2_-ratios (“∆”) of its values at all the tested concentrations with respect to the solvent controls. Then, we fit the feature’s dose response curve using either a standard log-logistic model or a constant model (“Materials and methods”), and determined its response value at the highest tested concentration based on the fitted curve (“∆_max_”). For brevity, all features that we mention in this article are referring to the ∆_max_ values of the respective features (Supplementary Material 2), and not the raw measured feature values, unless otherwise indicated. Among all the reference chemicals that did not cause NC, we found that BEAS-2B cells showed bioactivity (defined to be at least 30% change in at least 5% of the measured features) for 23 of the 28 chemicals (82.1%), but A549 showed bioactivity only for 22 of the 32 chemicals (68.8%) (Fig. 2d). These results show that BEAS-2B cells are phenotypically more responsive to the reference chemicals than A549 cells, irrespective of the pulmonotoxicity of the chemicals. This again agrees with the results from our earlier cell count analysis (Fig. 2b).

Then, we performed hierarchical clustering on the reference chemicals based on the obtained phenotypic feature values. For BEAS-2B, the chemicals could be divided into four major clusters (Fig. 2d). Cluster 1 consisted of only pulmonotoxic chemicals, including aristolochic acid I, carbamazepine, nitrofurantoin, paraquat, and tenofovir; and was characterized by large increases in several γH2AX features, such as mean γH2AX intensity at the chromosomal region (“*F*_a_”) and the number of γH2AX objects/foci (“*F*_b_”) (Fig. 2d and Supplementary Material 1—Fig. S4a). Cluster 2 consisted of a mixture of pulmonotoxic and non-pulmonotoxic chemicals, including cyclophosphamide, dipropylene glycol, and methotrexate; and was characterized by large increases in γH2AX object features, such as *F*_b_, but lower increases in γH2AX intensity features, such as *F*_a_. Cluster 3 also consisted of both pulmonotoxic and non-pulmonotoxic chemicals, including diacetyl and monocrotaline; and was characterized by large increases in γH2AX object features, such as *F*_b_, and actin intensity and object features, such as total cellular actin intensity (“*F*_c_”) and mean actin object size (“*F*_d_”). Finally, Cluster 4 consisted of all non-pulmonotoxic chemicals, which usually had low or no change in most of the measured features.

For A549 cells, we only obtained three major clusters (Fig. 2d). Cluster A consisted of mostly pulmonotoxic chemicals, and was characterized by large increases in many γH2AX and actin features, including *F*_a_, *F*_b_, and *F*_c_. However, only three out of the six chemicals in this cluster overlapped with Cluster 1 in BEAS-2B, namely, aristolochic acid I, nitrofurantoin, and paraquat. Cluster B consisted of a mixture of pulmonotoxic and non-pulmonotoxic chemicals, and was characterized by large increases in γH2AX object features, such as *F*_b_. Finally, cluster C consisted of mostly non-pulmonotoxic chemicals, which usually had low or no change in most of the features.

For both cell lines, we consistently found that the induction of γH2AX foci was not always associated with an increase in the γH2AX intensity (e.g., *F*_a_ vs. *F*_b_ in Fig. 2d). However, when both phenotypes occurred concurrently, the chemicals were more likely to be pulmonotoxic (e.g., Clusters 1 and A in Fig. 2d). Our results show that DDR, as indicated by *F*_a_ and *F*_b_, were induced by many pulmonotoxic chemicals in these two cell lines, which were originated from the bronchial or alveolar epitheliums, respectively. Many chemicals also caused changes in the actin cytoskeleton, but the effects appear to be non-specific to pulmonotoxic chemicals (especially for Cluster 3 in BEAS-2B cells, Fig. 2d). Therefore, features that are associated with the identified clusters may not be sufficient to predict in vivo pulmonary toxicity.

### γH2AX and DNA co-localization is predictive of pulmonotoxicity

**Fig. 3:**
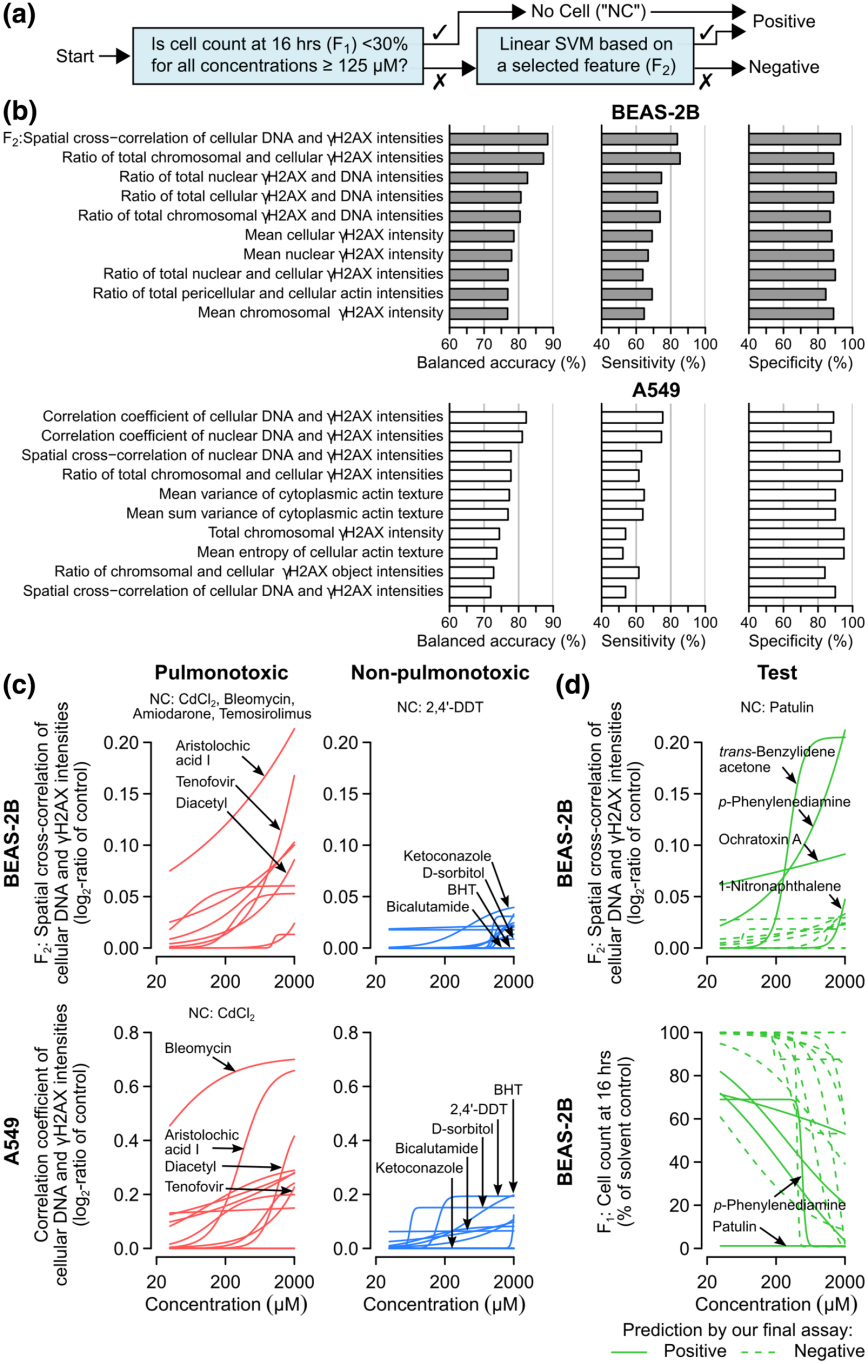
BEAS-2B cell count and co-localization of γH2AX and DNA are highly predictive of pulmonotoxicity. **a** Schematic showing a cascade classifier that uses, in succession, the cell count (*F*_1_) and a selected phenotypic feature (*F*_2_) at 16 h to classify pulmonotoxic and non-pulmonotoxic reference chemicals. **b** Bar charts showing the test balanced accuracies, sensitivities, and specificities of the top 10 phenotypic features estimated using the cascade classifier and a tenfold cross-validation procedure (“Materials and methods”). The feature with the highest estimated test balanced accuracy is the “spatial cross correlation of cellular DNA and γH2AX intensities in BEAS-2B cells” (*F*_2_). **c** Concentration–response curves of the reference chemicals (red = pulmonotoxic, and blue = non-pulmonotoxic) based on the best performing BEAS-2B (top) and A549 (bottom) phenotypic features (BHT = butylated hydroxytoluene). **d** Concentration–response curves of the test chemicals (green) based on *F*_1_ (bottom) and *F*_2_ (top) of BEAS-2B cells. A final assay based on *F*_1_ and *F*_2_ was trained on all the reference chemicals and applied to these test chemicals (solid lines = predicted to be positive, dashed lines = predicted to be negative by the final assay). (Color figure online)

During the second step of HIPPTox (Fig. 2c), we used a cascade classifier based on the changes in cell count (*F*_1_) and one of the measured phenotypic features to systematically identify the most predictive phenotypic feature (Fig. 3a). If a chemical was found to cause NC (i.e., < 30% cell count relative to the solvent control in all concentrations ≥ 125 µM), the chemical would be assigned to the “positive” class without any further evaluation. Chemicals that did not cause NC would be further evaluated using a linear support vector machine (SVM) (Ben-Hur et al. 2008) based on a selected phenotypic feature (*F*_2_), which would then assign the chemical to either the “positive” or “negative” class (Fig. 3a). We chose to use linear SVMs, because they produce continuous decision boundaries, which make the relationships between feature values and pulmonotoxicity easier to interpret. After the most predictive feature was identified, we also checked the values of the feature for those NC-causing chemicals at lower concentrations to determine if the feature may also cover the mechanisms of these chemicals.

After estimating the prediction performances of all the 166 phenotypic features using a ten-fold cross-validation procedure (“Materials and methods” and Supplementary Material 3), we found that most of the top performing features were related to DNA or γH2AX phenotypes, and less to cellular morphology or actin phenotypes (Fig. 3b). Specifically, the best feature of BEAS2B cells was “spatial cross correlation of cellular DNA and γH2AX intensities” (*F*_2_), which in combination with *F*_1_, gave 88.8% balanced accuracy, 84.6% sensitivity, and 93.0% specificity, while the best feature of A549 cells was “correlation coefficient of cellular DNA and γH2AX intensities”, which in combination with cell count, gave 82.4% balanced accuracy, 75.4% sensitivity, and 89.5% specificity (Fig. 3b). These two features are closely related to each other. Spatial cross correlation measures the similarity between the DNA and γH2AX staining patterns allowing possible spatial displacements between the two patterns; whereas correlation coefficient measures the degree of agreement or disagreement between the two patterns at the same subcellular locations (see “Materials and methods” for their definitions). The results suggest that, in BEAS-2B cells, activated γH2AX was mostly found at the vicinity of the DNA regions, whereas in A549 cells, it was mostly found at the DNA regions. We also compared the performance of different multi-feature sets automatically selected using a recursive feature elimination algorithm (Su et al. 2016), and found that inclusions of additional phenotypic features did not further increase the classification accuracy (Supplementary Material 1—Fig. S5). Thus, the best BEAS-2B features (*F*_1_ and *F*_2_) provide the highest accuracy and contain most of the discriminative information in our data sets.

The best BEAS-2B feature (*F*_2_) has both higher sensitivity and specificity than the best A549 feature. We compared the concentration–response curves based on these two features, and found that most of the pulmonotoxic chemicals (e.g., aristolochic acid I, bleomycin, diacetyl, and tenofovir) are more potent, while most of the non-pulmonotoxic chemicals (e.g., BHT, bicalutamide, and d-sorbitol) are less potent under the best BEAS-2B feature (Fig. 3c). Interestingly, the best features from both cell lines provided the same correct or incorrect predictions for most of the reference chemicals (Supplementary Material 1—Fig. S6). For example, both features failed to correctly predict the pulmonotoxicity or non-pulmonotoxicity of 2,4′-DDT, cyclophosphamide, and methotrexate. Only two chemicals, namely, nickel sulfate and BHT, were correctly predicted by the BEAS-2B feature, but not by the A549 feature. No chemical was correctly predicted only by the A549 feature (Supplementary Material 1—Fig. S6). These results suggest that pulmonotoxic chemicals may injure both cell lines, likely via the same mechanisms, but BEAS-2B cells have more sensitive and specific γH2AX responses than A549 cells. Therefore, BEAS-2B cells is a more preferable in vitro cell model for pulmonotoxicity prediction.

In BEAS-2B cells, there were five NC-causing chemicals, namely, 2,4′-DDT, amiodarone, bleomycin, cadmium(II) chloride, and temsirolimus (Fig. 2b). These chemicals were predicted to be “positive” without further evaluation of their phenotypic responses (Fig. 3a). We wondered if these chemicals also induced similar phenotypic responses as other pulmonotoxic chemicals. What were their *F*_2_ values at the highest tested concentrations before the induction of significant cell lost? We found that there were > 10% of BEAS-2B cells left after the cells were exposed to up to 31.25 µM 2,4′-DDT, 31.25 µM amiodarone, 62.5 µM bleomycin, or 500 µM temsirolimus. All tested concentrations of cadmium(II) chloride caused < 10% cells left, and thus, the chemical was not further analyzed. When the *F*_2_ values obtained under these conditions were compared to the *F*_2_ values of other chemicals obtained as described earlier, we found that 2,4′-DDT, amiodarone, and bleomycin caused similarly high increases of *F*_2_ as most other pulmonotoxic chemicals, but temsirolimus only mildly increased *F*_2_ (Supplementary Material 1—Fig. S7). Therefore, the first three chemicals may activate the same molecular pathways as other pulmonotoxic chemicals, but at much faster time scales. Unlike these three chemicals, temsirolimus may cause pulmonotoxicity through a different but currently unknown mechanism.

We decided to build a classifier for our final assay based on the changes in cell count (*F*_1_) and the best BEAS-2B feature (*F*_2_), and train the classifier using all the 33 reference chemicals. Then, the final assay was used to evaluate the 17 test chemicals with unknown or unclear human pulmonotoxicity (Fig. 3d and Supplementary Material 2). Interestingly, the final assay predicted 5 of the 17 test chemicals to be positive. They included patulin (a food toxin), which caused major cell lost in most of the tested concentrations (Fig. 3d, bottom); *p*-phenylenediamine, *trans*-benzylideneacetone (a favoring agent and fragrance additive), and ochratoxin A (a food toxin), which strongly activated *F*_2_ in the BEAS-2B cells (Fig. 3d, top); and 1-nitronapthalene (a common air pollutant), which had the weakest effect and thus the lowest confidence among the five positive predictions (Fig. 3d, top). Eleven of the test chemicals were predicted to be negative, including xylazine (a veterinary anesthetic agent and also an abused substance); thiamethoxam (an agricultural chemical); naphthalene and nicotine-derived nitrosamine ketone (NNK); 3-methylindole, β-myrcene, propyl paraben, diethyl phthalate, and dibutyl phthalate (all ingredients of consumer products); and 1,1-dichloroethylene and triethylene glycol (both industrial chemicals).

### Confirmation of DNA strand breaks

**Fig. 4:**
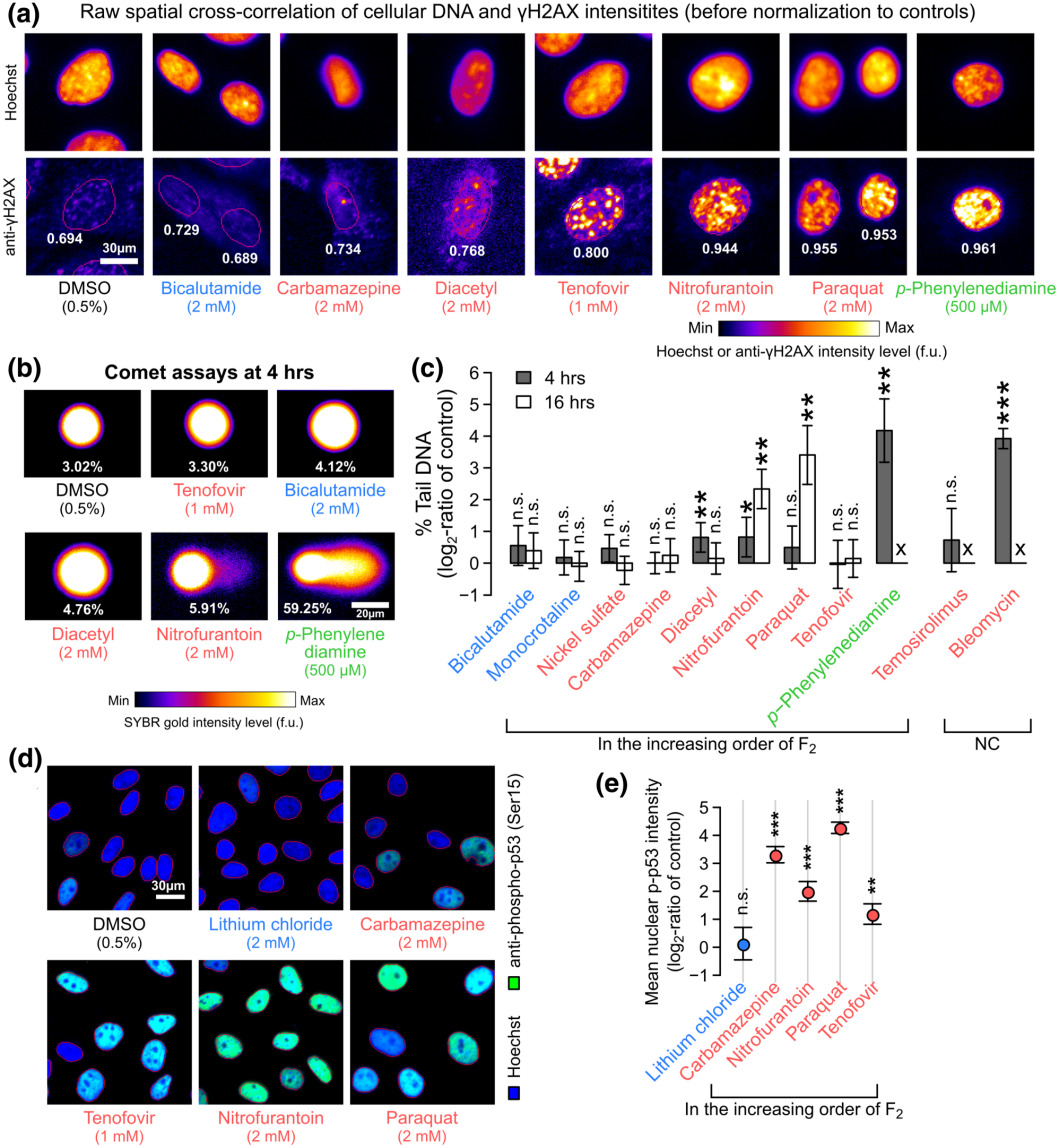
Pulmonotoxic chemicals may induce DDR pathway activations dependent or independent of DNA strand breaks. **a** Fluorescence microscopy images showing the staining patterns of Hoechst and anti-γH2AX in single BEAS-2B cells treated with the indicated chemicals for 16 h. Cells with raw *F*_2_ values (indicated below the cells) close to the average raw *F*_2_ values across all the cells under the same treatment conditions are shown (scale bar = 30 µm). To allow visual comparisons, all the shown images have been scaled to the same intensity ranges with respect to the solvent controls. **b** Fluorescence microscopy images showing the DNA spots obtained from the Comet assays of BEAS-2B cells treated with the indicated chemicals for 4 h. DNA spots with % tail DNA values (indicated below the patterns) close to the average % tail DNA values across all the spots obtained under the same treatment conditions are shown (scale bar = 20 µm). To allow visual comparisons, all the shown images have been scaled to the same intensity ranges with respect to the solvent controls. **c** Changes in the median % tail DNA values obtained from the Comet assays of BEAS-2B cells treated with the indicated chemicals or solvent controls for 4 or 16 h (bars = mean of the log_2_-ratio values obtained from at least three independent biological replicates, error bars = 95%-tile confidence intervals; X marks = ≤ 5% cells left). **d** Fluorescence microscopy images showing the staining patterns of Hoechst and anti-phospho-p53 (Ser15) in BEAS-2B cells treated with the indicated chemicals for 16 h. **e** Changes in the median nuclear phospho-p53 intensity levels of these cells quantified from the images with respect to the solvent controls (dots = mean of the median values, error bars = 95%-tile confidence intervals.) All *P* values shown in this figure are obtained from two-sided *t* tests and adjusted for false discovery rates (***P-adjusted ≤ 0.01, **P-adjusted ≤ 0.05, *P-adjusted ≤ 0.10)

What are the MoAs represented by *F*_2_ in BEAS-2B cells? The feature measures the spatial co-localization of the γH2AX and DNA markers (Fig. 4a and Supplementary Material 1—Fig. S8). In response to DNA damage, histone H2AX is phosphorylated on Ser139 (i.e., γH2AX) to recruit DNA repairing factors to the site of DNA damage (Rogakou et al. 1998; Paull et al. 2000). In parallel, tumor protein p53 (or “p53”), which plays a main role in activating DNA repair proteins, controlling cell cycle, and initiating apoptosis, is also being activated (Banin et al. 1998). Therefore, *F*_2_ is likely to represent the activation of this pathway, which is also collectively called the DDR pathway (Ciccia and Elledge 2010). We found that the mean nuclear γH2AX intensity had lower classification accuracy than *F*_2_ (78.0 vs. 88.8%; Fig. 3b), and thus, the feature may not fully represent the activation of the DDR pathway (Supplementary Material 1—Fig. S9). For example, diacetyl induced similar changes in mean nuclear γH2AX intensity, but higher changes in *F*_2_, than most of the non-pulmonotoxic chemicals, while monocrotaline was inducing opposite responses in these two features (Supplementary Material 1—Fig. S9). The result also demonstrates the advantage of using HIPPTox to automatically identify the most predictive phenotypic features.

An increase in *F*_2_ may indicate an increase in DNA strand breaks (DSBs) (Rogakou et al. 1998). However, there is an increasing awareness that other DSB-independent events, such as replication stress (Ward and Chen 2001), serum starvation (Lu et al. 2008), or even mitosis (McManus and Hendzel 2005), may also activate the DDR pathway. To confirm if the increase of *F*_2_ is due to DSBs, we performed single-cell gel electrophoresis (or “Comet”) assays (Singh et al. 1988) on ten reference and one test chemicals after 4 and 16 h of chemical exposure (Fig. 4b and Supplementary Material 1—Fig. S10). For each replicate, we identified ~ 300–500 DNA spots from the acquired images, and quantified the median percentage (%) of tail DNA for all the identified spots (“Materials and methods” and Supplementary Material 1—Table S4). Overall, we found that the increase in *F*_2_ was positively correlated to the increase in % tail DNA (Fig. 4b, c). Bleomycin and *p*-phenylenediamine induced strong DSBs at 4 h (50.8% and 57.0% tail DNA, respectively; Supplementary Material 1—Table S4), which lead to NC at 16 h. Nitrofurantoin and paraquat had slower effects, and only induced strong DNA strand breaks at 16 h (20.9 and 47.3% tail DNA, respectively; Supplementary Material 1—Table S4).

The rest of the pulmonotoxic reference chemicals show either low or no increase of DSBs, but only the effect of diacetyl at 4 h was significantly higher than the controls (FDR-adjusted *P* = 0.02; two-sided *t* test). Although the absolute magnitude of the increase was small (only 2.01% tail DNA, Supplementary Material 1—Table S4), most of the DNA spots under the control conditions were highly circular and homogenous (Supplementary Material 1—Fig. S10). For example, the standard deviation across all the biological replicates of DMSO controls was only 0.243%. Thus, the effect of diacetyl was still statistically significant. Interestingly, the DNA strand breaks induced by diacetyl decreased after 16 h (Fig. 4c), suggesting that the mild DNA damage may had been repaired.

### DSB-independent activation of the DDR pathway

Despite the relatively high induction of *F*_2_ by tenofovir, and to a lesser extent by carbamazepine, we found that these two chemicals caused very low or no increase in DSBs (Fig. 4b, c). Do they induce the DDR pathway? We performed another immunofluorescent assay to measure the nuclear level of phosphorylated p53 at Serine 15 (“p-p53”), which is known to be activated under DDR (Banin et al. 1998). Nitrofurantoin and paraquat were included as positive controls, and lithium chloride as a negative control. Interestingly, we found that both carbamazepine and tenofovir significantly increased the p-p53 level (FDR-adjusted *P* = 0.0021, and 0.0001, respectively; two-sided *t* test; Fig. 4d, e). Therefore, the DDR pathway (both γH2AX and p-p53) was activated by these two chemicals through DSB-independent mechanisms that are yet to be identified.

Our results show that many but not all pulmonotoxic chemicals induce mild or severe DSBs. However, *F*_2_ is sufficient to detect the effects of pulmonotoxic chemicals that cause DSBs or other currently unknown MoAs that activate the DDR pathway. Our results also agree with the previous observation that γH2AX is activated in lung sections from human asthma patients and house-dust-mite-exposed mice (Chan et al. 2016). All the results support the hypothesis that DDR is a general cellular-stress marker for pulmonotoxicity.

### Cell viability is a sensitive but non-specific marker

**Fig. 5:**
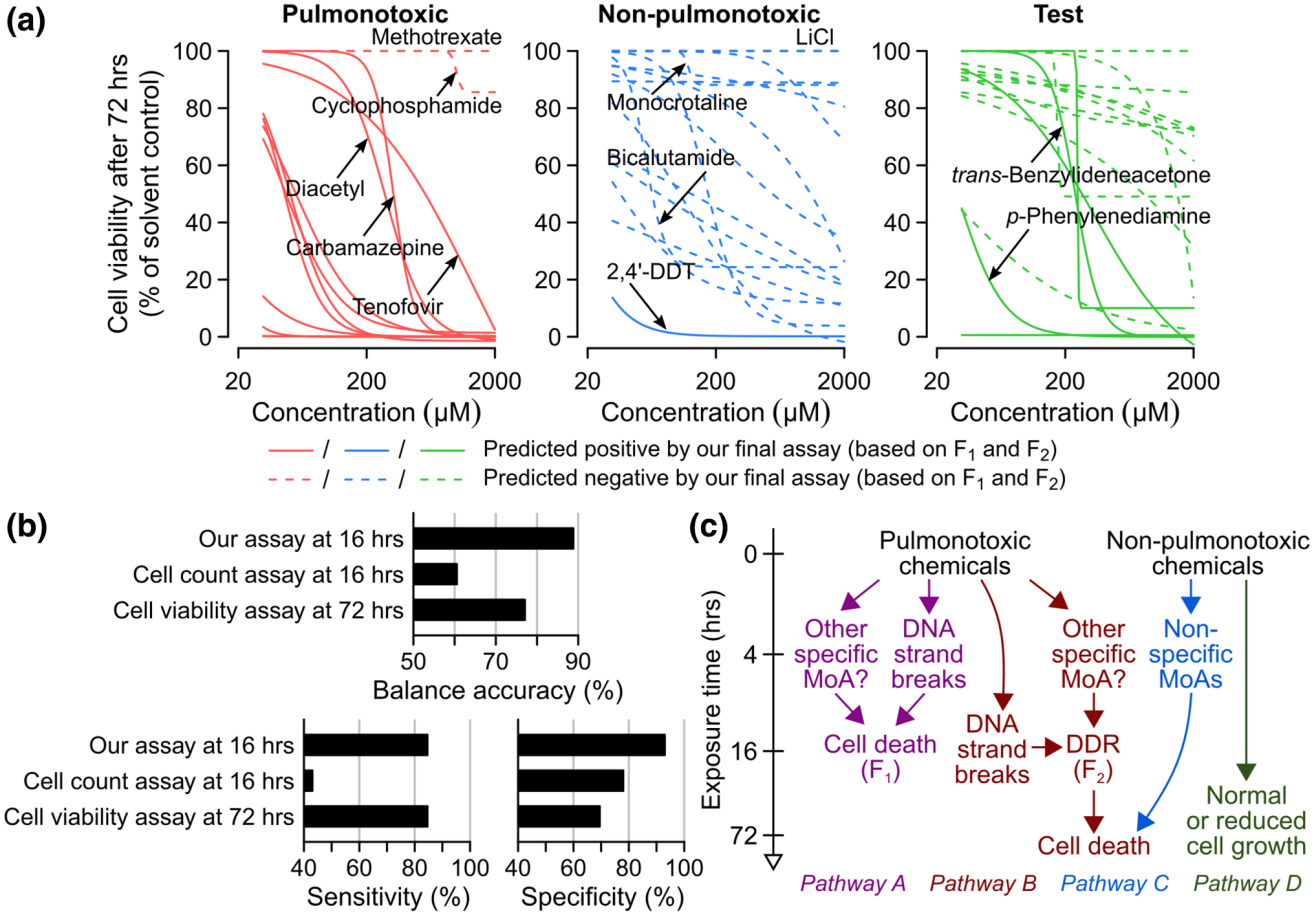
Our imaging-based assay is more predictive than cell count or viability assays. **a** Percentages of viable cells with respect to the solvent controls for all the 33 reference chemicals (red = pulmonotoxic, blue = non-pulmonotoxic) and 17 test chemicals (green). The values were measured using a standard cell-viability assay with BEAS-2B cells exposed to the chemicals for 72 h. Our final assay based on *F*_1_ and *F*_2_ and trained on all the reference chemicals was applied to all the reference and test chemicals (solid lines = predicted to be positive, dashed lines = predicted to be negative). **b** Test balanced accuracy, sensitivity, and specificity values of the three indicated assays in classifying the reference chemicals estimated using a tenfold cross-validation procedure. **c** Schematic showing the four major pathways activated by our reference and test chemicals. (Color figure online)

Does the induction of *F*_2_ eventually lead to cell death? How does our pulmonotoxicity assay compare to a standard cell-viability assay? We measured the viability of BEAS-2B cells treated with the 50 reference and test chemicals for 72 h using a standard resazurin assay (Fig. 5a). The same timepoint and/or assay were used in other previous in vitro studies (Lin and Will 2012; Sison-Young et al. 2017). We found that pulmonotoxic chemicals predicted to be “positive” by our final assay all caused cell death after 72 h (Fig. 5a). These chemicals include carbamazepine and tenofovir that induced very low or no DSBs at 4 and 16 h (Fig. 4c and Supplementary Material 1—Table S4). Therefore, the results suggest that BEAS-2B cells are sensitive to these chemicals, which may induce other DSB-independent MoAs. Interestingly, the two pulmonotoxic chemicals misdetected by our assay, namely, methotrexate and cyclophosphamide (Supplementary Material 1—Fig. S6 and S9), did not cause major cell death even after 72 h (Fig. 5a). Our final assay could not capture the effects of these two chemicals, whose effects may involve other molecular pathways or response dynamics that are not currently monitored by *F*_1_ and *F*_2_. Another possibility is that the chemicals may require bio-activation or other genetic/molecular factors not found in BEAS-2B cells or induced by our culturing conditions. This is supported by the previous findings that cyclophosphamide has little in vitro cytotoxic or alkylating activity until it is being metabolized, and the metabolism occurs mostly in the liver (Hill et al. 1972; Emadi et al. 2009).

We also found that most of the non-pulmontoxic chemicals induced noticeable cell loss (> 50%) at 72 h (Fig. 5a). For example, bicalutamide did not induce DNA damage or γH2AX activation at 16 h (Figs. 3c, 4c), but caused > 70% cell loss at ≥ 125 µM. Other non-pulmonotoxic chemicals, such as 2-hydroxypropyl-β-cyclodextrin and ketoconazole, also caused > 70% cell loss at concentrations as low as 125 µM. These results suggest that non-pulmonotoxic chemicals are likely to induce non-specific MoAs at longer exposure times. Interestingly, monocrotaline only caused a small decrease (< 20%) in the number of viable cells, despite the increase in mean nuclear γH2AX intensity caused by the chemical (Supplementary Material 1—Fig. S9), in agreement with our annotation and prediction that the chemical is not pulmonotoxic.

We performed a systematic comparison of the performances of assays based on either *F*_1_ and *F*_2_ at 16 h (our assay), *F*_1_ at 16 h only (an early cell count assay), or cell viability at 72 h (a later and more commonly used cell-viability assay) (Fig. 5b). We found that both cell count and viability assays could only achieve 60.5 and 77.1% balanced accuracy, 43.1 and 84.6% sensitivity, and 78.0 and 69.5% specificity, respectively. The results show that cell viability is a sensitive but non-specific in vitro marker for pulmonotoxicity. Furthermore, the increase in the sensitivity and the decrease in the specificity of these two assays were both positively related to the increase in chemical exposure time (Fig. 5b). Therefore, it is very difficult to find an optimal timepoint for cell-viability/death assays that could yield both maximum sensitivity and specificity. Due to the sensitivity of cell-viability/death assays, they may still be used to confirm the “positive” predictions made by our imaging-based assay, and determine if cells could recover from the toxic effects. However, they should not be used alone for pulmonotoxicity assessment.

## Discussion

In conclusion, we have developed an in vitro pulmonotoxicity assay based on *F*_1_ and *F*_2_ of BEAS-2B cells, which can be used to accurately and efficiently evaluate the potential pulmonotoxicity of soluble xenobiotics. Importantly, using HIPPTox and without making *a priori* assumptions about the MoAs of the reference chemicals, we have found that the in vitro lung-cell effects of the reference chemicals can be divided into four major sequences of molecular or cellular events (or “pathways”, Fig. 5c). The first two pathways are induced by the pulmonotoxic chemicals. “Pathway A” rapidly leads to cell death in 16 h or less. Some of the chemicals that activate this pathway, such as bleomycin and *p*-phenylenediamine, may induce significant DSBs as early as 4 h (Fig. 4c). Other chemicals, such as temsirolimus, may cause cell death via a currently unknown but likely DDR-independent mechanism (Supplementary Material 1—Fig. S7). “Pathway B” takes relatively longer times to activate DDR (including γH2AX and p53), and then induce cell death. Some of the chemicals that activate this pathway, such as diacetyl, nitrofurantoin and paraquat, may cause DSBs; but others, such as carbamazepine and tenofovir, do not cause noticeable DSBs (Fig. 4c). The slower dynamics of these chemicals may be due to the requirement of their bio-activations or other intermediate reactions in the lung cells. The DSBs caused by some of these chemicals, such as diacetyl, may be mild and “recoverable”, but eventually the exposed cells would still die from the insults (Fig. 5a). Many of the chemicals that activate these two pathways, including carbamazepine, diacetyl, nitrofurantoin, *p*-phenylenediamine, and tenofovir are not previously known to activate DDR in the lung cells. Further studies are required to determine if the observed phenomena are direct or indirect effects of these chemicals, which may be due to the generation of oxidative stress (Chan et al. 2016). Indirect DDR activations may also be facilitated by the binding to nuclear receptors or transcription factors, such as the aryl hydrocarbon receptor (AhR), that may shuttle reactive chemicals or their metabolites from the cytoplasm into the nucleus (Park et al. 2009). DDR activation is commonly used as a marker for genotoxicity; but in agreement with other previous reports (Chan et al. 2016, Su et al. 2016), our results suggest that it may also be a cellular-stress marker, and activated by chemicals that do not necessary or directly cause DNA damages.

The other two pathways are induced by the non-pulmonotoxic chemicals (Fig. 5c). “Pathway C” can cause cell death without activating the DDR pathway, and is activated, for example, by bicalutamide (Figs. 2b, 4c, 5a). The effects of the chemicals that activate this pathway are usually positively correlated with the chemical exposure times or concentrations. However, some of them, such as ciprofloxacin and nystatin at 500 µM, caused noticeable reduction in cell count (to ~ 30.9 and 0% relative to solvent controls, respectively) as early as 16 h. The existence of this pathway is a major reason for the low specificity of a cell-viability assay. “Pathway D” do not cause major cell death, even when activated by chemicals at the highest tested concentrations and timepoints. Monocrotaline and lithium chloride are two examples of chemicals that activate this pathway (Fig. 5a, middle). The lack of cell death effects does not necessary imply null cellular effects. For example, we found that monocrotaline-induced dramatic changes in the actin cytoskeleton and increase of mean nuclear γH2AX level in BEAS-2B cells (Fig. 2d and Supplementary Material 1—Fig. S9). The inclusion of chemicals that can activate Pathways C and D as negative reference chemicals during the development of in vitro toxicity assay was critical to ensure that specific phenotypic endpoints were being identified and used for the final assay. In many potential applications of our assay, such as screening of chemical candidates during drug or other chemical product developments, assays with higher specificity are more desirable to ensure that candidates with desired efficacy or other beneficial properties are not being removed early on the process.

The adverse outcome pathway (AOP) is a toxicology knowledge framework for chemical risk assessment based on mechanistic reasoning (Ankley et al. 2010). At the time of preparing this report, there was no approved AOP for any lung adverse effect. Five AOPs were proposed or under development for lung fibrosis or irritation (Supplementary Material 1—Table S5). While cellular inflammation was proposed to be a key event in many of these proposed lung AOPs, none of them included oxidative-stress or DNA-damage responses as key events. However, the interplay between oxidative-stress response and inflammation is known to play an important role in the pathogenesis of many xenobiotic-induced lung diseases (Tuder and Petrache 2012). For example, cigarette smoke-induced oxidative stress may induce a protein called “Regulated in Development and DNA-damage-responses 1” (Redd1/Rtp801), which is required and sufficient to activate the nuclear factor-κB (NF-κB) signaling pathway in lung cells (Yoshida et al. 2010). Increase in the γH2AX level was also found in the lung lysates of human asthma patients and house-dust-mite-exposed mice (Chan et al. 2016). Together with our results, all of these findings suggest that oxidative-stress or DNA-damage responses may be a key event, either upstream or downstream of cellular inflammation, in the AOPs for lung adverse effects.

Currently, our assay may be used to predict the potential pulmonary hazards of soluble chemicals. Measurements or modeling of the exposure and bioavailability of the predicted “positive” chemicals in the lungs are still required to fully assess the safety and risk of these chemicals. The toxicokinetics of these chemicals may be estimated, for example, using the High-throughput Toxicokinetics (HTTK) tools developed by the US Environmental Protection Agency (Wetmore et al. 2015). Furthermore, our assay currently does not cover vaporized or particulate compounds, which are two other major sources of potentially pulmonotoxic chemicals. However, HIPPTox is a general approach and can be applied to other more complex in vitro lung models that can better mimic the exposures and uptakes of these types of chemical compounds. HIPPTox can also be applied to other non-lung-originated cell types, or even non-imaging molecular or phenotypic profiling methods, such as RNA sequencing or mass spectrometry. It is a general and powerful approach that can greatly accelerate the development of predictive alternative assays.

## Materials and methods

### Chemical preparation and quality control

The full list of candidate chemicals and their solvents and sources are provided in Supplementary Material 1—Table S1. All chemicals were prepared either in DMSO or in ethanol at a stock concentration of 400 mM, or in water at a stock concentration of 20 mM. Four of these chemicals, namely, aristolochic acid I, bleomycin, nystatin, and tenofovir, were found to yield highly viscous suspensions in DMSO, which made them difficult to be transferred by pipetting. To overcome this, we further diluted these four chemicals to a stock concentration of 200 mM. Therefore, in the final experiments, the highest test concentration for all chemicals is 2 mM, except for these four chemicals, 1 mM.

To evaluate the suitability of the candidate chemicals for our imaging assay, we performed three different sets of chemical quality-control (QC) experiments. First, we checked the solubility of all the 60 chemicals by diluting them in bronchial epithelium cell growth medium (BEGM; #CC-3170; Lonza, Basel, Switzerland) down to 1 or 2 mM (see above). The chemicals that were not fully soluble were sonicated for at least 5 min and left to stand for ~ 15 min. After which, chemicals that yielded clear solutions with undissolved compounds settling down at the bottom of the sample vials were considered to be “insoluble”. They include barium sulfate, ferrocene, gallium(III) oxide, and iron(III) oxide. Second, we checked the autofluorescence of the chemicals. Please refer to the following section on “Autofluorescence assay and analysis” for more detailed information. Four candidate chemicals were found to have unacceptably high autofluorescence levels, namely, benzo[α]pyrene, manganese(II) acetate, manganese(II) chloride, and vancomycin hydrochloride (Supplementary Material 1—Fig. S1). Finally, we also visually inspected the fluorescence microscopy images of cells treated with these chemicals for obvious imaging artifacts, such as the formation of highly fluorescent objects/speckles at the background (i.e., non-cellular regions), which may be due to the binding of the chemicals to the coating materials or the assay plates. Two candidate chemicals, namely, cotinine, and 3,3′,5-triiodo-l-thyronine, were removed. In summary, we found a total of 50 candidate chemicals that pass all the chemical QC checks and are suitable for our imaging assays (Supplementary Material 1—Table S1).

### Pulmonotoxicity annotations

For 33 of the candidate chemicals, we could obtain published human information from expert reviews/reports, clinical studies, post-marketing safety surveillance, poisoning-incident, reports, and/or epidemiological studies. The following databases were used to search for these publications: Agency for Toxic Substances and Disease Registry (ATSDR, https://www.atsdr.cdc.gov), Centres for Disease Control and Protection National Biomonitoring Programme (https://www.cdc.gov/biomonitoring), US National Library of Medicine (NLM)’s DailyMed (https://dailymed.nlm.nih.gov/dailymed), National Toxicology Program Technical Report (https://ntp.niehs.nih.gov), and US NLM Hazardous Substances Data Substances Data Bank (https://toxnet.nlm.nih.gov/newtoxnet/hsdb.htm). For pharmaceuticals with safety surveillance data, we only assigned them to the “pulmonotoxic” class if their adverse pulmonary effects were found in > 1% of patients. Detail descriptions and references that we used to assign the annotations are provided in Supplementary Material 1—Table S2.

### Cell culture

We expanded BEAS-2B and A549 cells (#CRL-9609 and #CCL-185, respectively; ATCC, Manassas, USA) from frozen stocks for at least two passages before using them for our experiments. We maintained BEAS-2B cells in BEGM (#CC-3170; Lonza, Basel, Switzerland), which was pre-supplemented with bovine pituitary extract, hydrocortisone, human epidermal growth factor, epinephrine, transferrin, insulin, retinoic acid, and triiodothyronine, as recommended by the manufacturer, and 1% penicillin/streptavidin (#15140122; Gibco, Waltham, USA). We maintained A549 cells in Roswell Park Memorial Institute (RPMI; #11875119; Gibco, Waltham, USA) supplemented with 10% fetal bovine serum (#SV30160; Hyclone, Logan, USA) and 1% penicillin/streptavidin. All cells were maintained at 37 °C and 5% CO_2_. All the BEAS-2B cell culture flasks were pre-coated with a coating medium, consisting of bronchial epithelium cell basal medium (BEBM; #CC-3171; Lonza, Basel, Switzerland), 0.01 mg/mL human fibronectin (#29011; Santa Cruz Biotechnology, Dallas, USA), 0.01 mg/mL bovine serum albumin (#7906; Sigma Aldrich, St. Louis, USA) and 0.03 mg/mL calf skin collagen type I (#8919; Sigma Aldrich, St. Louis, USA) for 60 h at 37 °C, which was removed before cell seeding. We also randomly performed mycoplasma tests on both cell lines throughout the course of our study using a PCR-based mycoplasma detection kit (#20-700-20; Biological Industries, Cromwell, USA), and found no mycoplasma contamination.

### Chemical exposure

We seeded the cells into black 384-well glass-bottom plates (#164586; NUNC, Waltham, USA) at 3 × 10^3^ cells/well using an automated liquid dispenser (Multidrop Combi reagent dispenser; Thermo Fisher Scientific, Vantaa, Finland). For BEAS-2B cells, the plates were pre-coated with BEBM coating media containing additional 5 µg/mL human fibronectin. For A549 cells, the plates were pre-coated with 2 µg/mL human fibronectin in sterile phosphate-buffered saline (PBS). All plates were coated for 60 h at 37 °C, and the coating medium was removed before cell seeding. After allowing the cells to grow on the plates for 48 h, we treated them with the reference chemicals at 2000, 1000, 500, 250, 125, 62.5, and 31.3 µM; and for aristolochic acid I, bleomycin, nystatin, and tenofovir, at 1000, 500, 250, 125, 62.5, 31.3, and 15.7 µM (see “Chemical preparation and quality control”). In every assay plate, wells with cells treated with 10 µM doxorubicin (positive control for the γH2AX antibody), 0.5% DMSO, 0.5% ethanol, or 10% water (all solvent controls) were also included. Four technical replicates were performed for each tested chemical and concentration.

### Fluorescent marker staining

After 16 h of chemical exposure, we fixed the cells with 4% paraformaldehyde (#28906; Pierce, Waltham, USA) in PBS. The cells were permeabilized with TBS-T, which consist of tris-buffered saline (TBS) with 0.1% triton X-100 (#H5142; Promega, Madison, USA). This was followed by 1 h blocking with 20% bovine serum albumin (#A7906; Sigma, St. Louis, USA) in TBS-T. We then incubated the cells with 1:500 rabbit monoclonal antibody to γH2AX (Ser139) (#9718; Cell Signaling Technology, Danvers, USA) overnight at 4 °C, and blocked the cells using the same blocking buffer for 15 min. For cells exposed to the chemicals that passed the green channel autofluorescence test (see “Autofluorescence assay and analysis”), they were incubated with 1:500 goat anti-rabbit secondary antibody conjugated to Alexa Fluor 488 (#A11034; Invitrogen, Waltham, USA), and 2.5 µg/mL deep red Whole Cell Stain (#H32721; Molecular Probes, Waltham, USA). For cells exposed to the chemicals that failed the green channel autofluorescence test, but passed the far-red autofluorescence channel test, they were incubated with 1:500 goat anti-rabbit secondary antibody conjugated to Alexa 647 (#A21245; Invitrogen, Waltham, USA), and 2.5 µg/mL green Whole Cell Stain (H32714; Molecular Probes, Waltham, USA). All secondary antibody incubations were performed at room temperature for 1.5 h in the dark. Lastly, we stained the cells with 0.3 µg/mL nucleic acid stain (#H1399; Molecular Probes, Waltham, USA), and 1:500 fluorescently labeled phalloidin (#13054; Cell Signaling Technology, Danvers, USA) for 15 min at room temperature, followed by washing the cells with TBS before imaging. For the p53 imaging assay, similar cell staining procedures were performed, except that we stained the cells with 1:400 mouse anti-phospho-p53 (Ser15) primary antibody (#9286, Cell Signaling Technology, Danvers, USA) and 1:500 goat anti-mouse secondary antibody conjugated to Alexa 488 (#A21121; Invitrogen, Waltham, USA).

### High-throughput imaging

We imaged the plates automatically using an inverted fluorescence microscope (Axio Observer Z1; Zeiss, Oberkochen, Germany) equipped with a 20× objective (0.8 NA), a laser auto-focus system (Definite Focus; Zeiss, Oberkochen, Germany), and a scientific charge-coupled-device (CCD) camera (CoolSNAP HQ2; Photometrics, Tuscon, USA). We imaged four fluorescence channels: Ex: 365/Em: 465 nm (the “blue channel”; Zeiss filter set 49), Ex: 470/Em: 525 nm (the “green channel”; Zeiss filter set 38), Ex: 545/Em: 605 nm (the “red channel”; Zeiss filter set 43) and Ex: 628/Em: 692 nm (the “far-red channel”; Semrock Cy5-4040B). The exposure times were ~ 30–50 ms (the blue channel), ~ 1–3 s (the green channel), 500 ms (the red channel), and ~ 1–3 s (the far-red channel). Within each well, four images at different locations were acquired and saved in 16-bit TIFF format.

### Image processing and analysis

All images were corrected using the “rolling ball” algorithm implemented in ImageJ (v1.51j8; NIH, USA). Cell segmentation and feature measurements were performed using the cellXpress software (v1.4.3; Bioinformatics Institute, Singapore) (Laksameethanasan et al. 2013). We extracted 65 texture features, 36 intensity features, 29 intensity ratio features, 18 correlation features, 17 morphology features, and cell count from the images. The detail list of features and their markers is shown in Supplementary Material 1—Table S3. The definition of the texture features can be found in our previous report (Su et al. 2016). The mathematical definitions of spatial cross correlation and correlation coefficient (TM_CCORR_NORMED and TM_CCOEFF_NORMED, respectively) can be found in the online documentation of OpenCV Library (https://docs.opencv.org), in which the cellXpress software is based on.

### Autofluorescence assay and analysis

We treated BEAS-2B cells with the chemicals at 2 mM (except for BHT, 3-methylindole, ochratoxin A at 500 µM; *p*-phenylenediamine at 250 µM; β-myrcene and aristolochic acid I at 125 µM; and amiodarone, bleomycin, cadmium chloride, and patulin at 15.7 µM) for 16 h. These lower concentrations were used, because the associated chemicals caused noticeable cell lost at higher concentrations. The cells were fixed and permeabilized as described above, stained with 0.3 µg/mL nucleic acid stain for 5 min in the dark, and finally washed with TBS before imaging. We imaged the cells as described above, except that the exposure times were set at 5000 ms for the green, red, and far-red channels. Since the cells were not stained in these three channels, the signals detected in these channels were likely due to the autofluorescence of the cells and/or the exposed chemicals.

The foreground-to-background fluorescence intensity ratios (FBRs) were measured using the cellXpress software, which automatically identified the foreground (cellular) and background (non-cellular) regions from each acquired image based on the nucleic acid stains. Then, we measured the 99.9%-tile and 50%-tile intensity values of all the foreground and background pixels, respectively. The log_2_-ratio of these two quantities is called the FBRs. For each chemical and fluorescence channel, the median FBR value across four replicates was determined, and is shown in Supplementary Material 1—Figure S1. To determine the maximally acceptable FBRs, we also quantified the FBRs for BEAS-2B cells under three positive-control conditions: (a) treated with 1 mM tenofovir, stained with Alexa-488-labeled anti-γH2AX antibody, and imaged at the green channel for 2000 ms; (b) treated with 0.5% DMSO, stained with phalloidin, and imaged at the red channel for 500 ms; or (c) treated with 1 mM aristolochic acid I, stained with Alexa-647-labeled anti-γH2AX antibody, and imaged at the far-red channel for 4000 ms. We chose the maximally acceptable autofluorescence FBRs to be 1/3 of the FBR values obtained for these three conditions (shown as red lines in Supplementary Material 1—Figure S1). Based on these results, we determined the fluorescent dyes that can be used to stain the cells (see “Fluorescent marker staining”).

### Cell-viability assay and analysis

We seeded BEAS-2B cells into black 384-well optical bottom plates (#3712; Corning, Corning, USA) at 4 × 10^2^ cells/well using an automated liquid dispenser. Plates were pre-coated with BEBM coating media for at least 60 h at 37 °C. After 48 h, we treated the cells with the 50 reference and test chemicals at the same concentrations as the imaging assay. Wells with no cell (positive controls for complete cell death) or BEAS-2B cells treated with 0.5% DMSO, 0.5% ethanol, or 10% water (solvent controls) were also included in each plate. At 1 h before measurement, we incubated the cells with 1.3 mg/mL of resazurin sodium salt (#14322; Cayman Chemical, Ann Arbor, USA) in sterile PBS at 37 °C and in the dark. Then, we measured the fluorescence signal using a fluorescent plate reader at Ex: 540/ Em: 590 nm.

For the cell-viability assay, we performed five technical replicates per chemical, and used a more stringent assay QC procedure. First, for each plate, we determined the mean background level from the positive control wells without cell, and subtracted this background level from the measurements from all other wells in the same plate. Then, for each chemical, we checked the coefficient of variation (CoV) of the solvent-control wells associated with the chemical. If the CoV was > 30%, we identified the wells that contributed to the most to the CoV, and discarded either one or two wells until the CoV became < 30%. Then, the median of the remaining control wells was taken as the average control cell-viability value. The same QC procedure was repeated for the treatment wells for the chemical at each treatment concentration. However, we allowed the remaining treated wells for the chemical to have CoV > 30% if their median cell-viability value was less than 20% of the median cell-viability value from the corresponding solvent-control wells. The rationale is that the cell death response to a pulmonotoxic chemical may be highly heterogeneous. For each chemical, we allowed at most one QC-failing concentration. If we could not find at least six concentrations (or data points) for the chemical, we would completely discard the collected data and perform new experiments for the chemical. Out of the 50 chemicals, only dibutyl phthalate required such an experimental repeat.

### Concentration–response-curve estimation and selection

To evaluate the change in a phenotypic feature or cell viability induced by a chemical at concentration $x$, $\Delta (x)$, we first computed the baseline response $\tilde {r}{\text{(0)}}$, which is taken as the median across all the responses from the solvent-control wells associated with the chemical. Then, $\Delta (x)={\log _2}\left( {{{\tilde {r}(x)} \mathord{\left/ {\vphantom {{\tilde {r}(x)} {\tilde {r}(0)}}} \right. \kern-0pt} {\tilde {r}(0)}}} \right)$, where $\tilde {r}(x)$ is the median response of the feature across all the cells in wells treated the chemical in concentration $x$. The measured $\Delta (x)$ values were used to build continuum models for the phenotypic feature as functions of the chemical concentrations. For all phenotypic features, except for cell count and cell viability, these models are

$${\text{Model}}\;1{:}\;{\Delta _{{\text{model}}}}(x)=\alpha - \frac{\alpha }{{1+\exp \left( {\beta \left( {\log x - \log \gamma } \right)} \right)}},$$

$${\text{Model}}\;2{:}\;{\Delta _{{\text{model}}}}(x)=\frac{{\alpha ^{\prime}}}{{1+\exp \left( { - \beta ^{\prime}\left( {\log x - \log \gamma ^{\prime}} \right)} \right)}},{\text{ and}}$$

$${\text{Model}}\;3{:}\;{\Delta _{{\text{model}}}}(x)=0,$$

where $\alpha$, $\beta$, $\gamma$, $\alpha ^{\prime}$, $\beta ^{\prime}$, and $\gamma ^{\prime}$ are all parameters estimated by least-square-error minimization of the models to the measured $\Delta (x)$ values. We used the “drc” library (v3.0-1) under the R environment (v.3.4.1; The R Foundation, Vienna, Austria) to fit each of the models. In qualitative terms, Model 1 represents a log-logistic sigmoidal concentration–response curve where increasing concentration of an applied chemical increases the value of the phenotypic feature relative to the controls; Model 2 represents a similar curve decreasing the value of the phenotypic feature relative to the controls; and Model 3 is the null model, where application of the chemical within the tested concentration levels does not alter the value of the phenotypic feature relative to the controls. We modeled cell count and cell-viability data slightly differently, in that $\Delta (x)$ for these features was not log_2_-transformed prior to curve-fitting, Model 1 was not used, and Model 3 was replaced with ${\Delta _{{\text{model}}}}(x)=\alpha ^{\prime\prime}$, which is a vertical linear model with a *y*-intercept of $\alpha ^{\prime\prime}$.

We evaluated the relative quality of the models by computing their Akaike information criteria (AIC) (Akaike 1974):

$${\text{AIC}}=2\left( {D - \log \left( {\frac{{\sum {{{\left( {{\varepsilon _k}} \right)}^2}} }}{m}} \right)} \right),$$

where $D$ is the number of degrees of freedom in a model, ${\varepsilon _k}$ is the residual error for data point $k$, and $m$is the number of data points (or tested concentrations). After determining the best model using the AIC, we estimated maximum response values, ${\Delta _{\hbox{max} }}$, for each feature and compound from their selected concentration–response models. In our study, ${\Delta _{\hbox{max} }}$ are equal to the normalized responses estimated at 2000 µM, this being equal to the highest sampled experimental concentration. The final result was a 166 (features) × 50 (chemicals) matrix of ${\Delta _{\hbox{max} }}$ values (Supplementary Material 2), which were used for training and testing the classifiers.

### Supervised classification and performance estimation

We used the linear SVM (Ben-Hur et al. 2008) to predict xenobiotic-induced pulmonary toxicity, and a stratified tenfold cross-validation procedure (Su et al. 2016) to estimate the prediction performance of our phenotypic features. A linear SVM has a key parameter, $C$, which controls the cost of misclassification on the training data. During each fold of the cross validation, we automatically determine the optimum classifier parameter using a grid search for $C=\left\{ {{{10}^0},\,{{10}^1},\,{{10}^2},\,{{10}^3},\,{{10}^4},\,{{10}^5}} \right\}$. Before data classification, each feature was normalized to the same range [− 1, 1]. As described previously (Su et al. 2016), to ensure the training and test data sets were independent to each other, the feature normalization coefficients and classifier parameter were always estimated based on the training data sets only, but applied to both training and test data sets. We used the LiblineaR() function in the “LiblineaR” library (v2.10-8) under the R environment, and set the *bias* parameter of the function to 1.

### Comet assay

We used an alkali Comet assay to quantify the extent of DNA strand breaks (DSBs) (Singh et al. 1988). We seeded BEAS-2B cells into 6-well plates (#3506; Costar™, Corning, New York, USA) at ~ 80–100 × 10^3^ cells/well. The plates were pre-coated with BEBM coating media for 60 h at 37 °C. After 48 h, we treated the cells with either 2 mM paraquat, 2 mM nitrofurantoin, 2 mM carbamazepine, 2 mM lithium chloride, 1 mM tenofovir, 0.5% DMSO, 10% water (both solvent controls), or 0.1% hydrogen peroxide (positive control). The cells were harvested 4 or 16 h after the chemical exposure (except for hydrogen peroxide, which was applied to the cells for only 15 min) using trypsin EDTA (#L11-003; PAA, Pasching, Austria) in cold sterile PBS. The cell suspension was adjusted to a density of ~ 150 × 10^3^ cells/mL, and mixed homogenously in a ratio of 1:5 with 1% low-melting-point agarose gel (#16520100; Invitrogen, Waltham, USA) in distilled water maintained in a 40 °C water bath. Then, we pipetted a thin layer of cell suspension onto a gel bond film (#53759; Lonza, Basel, Switzerland), and left the cell-suspension-agarose slides to set for 15 min at room temperature. We lysed the cells for 2–3 h at 4 °C in a freshly prepared lysis buffer containing 1.2 M sodium chloride (#37144; Kanto Chemical, Tokyo, Japan), 100 mM EDTA disodium salt (#H5032; Promega, Madison, USA), and 0.1% *N*-lauroylsarcosine sodium salt (#61747; Sigma Aldrich, St. Louis, USA) in distilled water, with pH adjusted to 13. After which, the slides were rinsed with an electrophoresis buffer containing 2 mM EDTA disodium salt in distilled water, with pH adjusted to 12.3. We then performed gel electrophoresis with the electrophoresis buffer for 25 min at a voltage of 0.6 V/cm, followed by neutralization with distilled water for 10 min. The slides were stained for 20 min with 1:2500 nucleic acid gel stain (SYBR Gold, #S11494; Invitrogen, Waltham, USA) and washed with distilled water before imaging. We used the same imaging procedures as described above to image at least 30 random locations within each slide, and obtained several hundred DNA spots from the images. For each chemical, we performed at least three biological replicates using different batches of cells.

### Quantification of DNA strand breaks

The images obtained from the Comet assays were first pre-processed to facilitate automated detection and quantification of DNA spots. We scripted a macro in Fiji (http://imagej.net/Fiji/Downloads) (Schindelin et al. 2012) to accomplish this task. First, we removed bright speckles in the images with a 3 × 3 median filter (the “Despeckle” command in Fiji), and smoothed the resulting images with a Gaussian blur filter (Gaussian Blur 3D plugin in Fiji) with the parameters: $x=5,\,\,y=5,\,\,z=0.$ Then, we applied a Laplacian-of-Gaussian (LoG) filter (Sage et al. 2005) with the parameters: ${\sigma _x}=35,\,\,{\sigma _y}=35,\,\,{\sigma _z}=0.$ The response of the LoG filter is high on the stained DNA spots, but low on background regions with high intensity values, which usually correspond to imaging artifacts. To isolate all the DNA spots from the original images, we binarized the resulting response images with an automatic thresholding algorithm. These pre-processing and background subtraction steps helped us to minimize interference from the uneven backgrounds.

We detected and quantified the DNA spots on the pre-processed images using the OpenComet software (v1.3.1, http://www.cometbio.org) (Gyori et al. 2014). The software automatically detects DNA spots by exploiting shape information, such as the expected convex shape and symmetricity of a comet (Gyori et al. 2014). It further analyzes an arbitrary intensity profile along the horizontal axis of the detected DNA spots, and marks the position of the largest intensity change as the demarcation between the head and tail of a comet. After the automated detection, we manually inspected the results, and removed misdetected DNA spots, such as neighboring spots with big tails that were wrongly merged together, irregular sized and shaped debris from the background, or out-of-focus DNA spots. We removed around ~ 10–20% of the identified DNA spots, and obtained ~ 400–500 usable spots for each treatment condition.

The OpenComet software can quantify the continuous distributions of different comet parameters, which are more informative and systematic than visual scoring (Kumaravel et al. 2009). Specifically, we chose to use the percentage (%) of tail DNA as a readout for DSBs. This parameter measures the percentage of total DNA intensity in the tail region compared to the total intensity of the entire DNA spot (Møller et al. 2014). For each treatment condition, we took the median of % tail DNA values obtained from all the DNA spots. Three or more biological replicates (on different batches of cells) were performed for each treatment condition, resulting in a total of 74 Comet assays. By inspecting the distributions of % tail DNA of all the replicates, we identified and removed 5 clear outliers from the 74 experiments. Finally, for each treatment condition, we determined the mean of the median % tail DNA values from all the remaining replicates (Supplementary Material 1—Table S4).

### Multi-dimensional scaling plot

To compare the compounds in the chemical structure space, we used the ChemmineR library (v2.30.0) to compute the pairwise Tanimoto coefficients between the structures of all the reference compounds. To compare the compounds in the phenotypic feature space, we first scaled all the phenotypic features to the same range [0, 1], and then computed the pairwise Euclidean distances between the feature values of all the reference compounds. Finally, we used the cmdscale() function in the R environment to generate the multi-dimensional scaling plots.

### Statistical analysis

The log_2_-ratio between the mean response of a set treated replicates and the mean response of a set of control replicates is equivalent to the difference between the respective values after log_2_ transformation: $\Delta (x)={\log _2}\left( {{{r(x)} \mathord{\left/ {\vphantom {{r(x)} {r(0)}}} \right. \kern-0pt} {r(0)}}} \right)={\log _2}\left( {r(x)} \right) - {\log _2}\left( {r(0)} \right)$. Thus, under the null hypothesis, $\Delta (x)=0$; and the statistical significance of the change, including its *P* value and 95% confidence interval, can be estimated using a standard two-sample *t* test on the log_2_-transformed data. To correct for multiple hypothesis testing, all the *P* values from the same experiment were corrected for false discovery rate using the Benjamini and Hochberg procedure (Benjamini and Hochberg 1995). We used the t.test() and p.adjust() functions in the R environment to perform the analysis.

## Electronic supplementary material

Below is the link to the electronic supplementary material.

Supplementary material 1 (PDF 1965 KB)

Supplementary material 2 (XLSX 174 KB)

Supplementary material 3 (XLSX 29 KB)
