# Supplementary material for: Building predictive in vitro pulmonary toxicity assays using high-throughput imaging and artificial intelligence
Source: Arch Toxicol. 2018 Apr 28;92(6):2055–75. doi: 10.1007/s00204-018-2213-0 (PMC6002469; doi:10.1007/s00204-018-2213-0)
Supplement: Supplementary file 1 — Supplementary material 1 (PDF 1965 KB) [file 204_2018_2213_MOESM1_ESM.pdf]

SUPPLEMENTARY MATERIAL 1

Building predictive *in vitro* pulmonary toxicity assays using high-throughput imaging and artificial intelligence

Jia-Ying Joey Lee, James Alastair Miller, Sreetama Basu, Ting-Zhen Vanessa Kee, and Lit-Hsin Loo*

Bioinformatics Institute, Agency for Science, Technology, and Research, 30 Biopolis Street, #07-01 Matrix, Singapore 138671, Singapore

* Correspondence should be addressed to LHL: loolh@bii.a-star.edu.sg

Figure S1. **Detection of autofluorescent chemicals**

Figure S2. **Benzo[α]pyrene is strongly autofluorescent in our three imaging channels**

Figure S3. **Examples of full-frame microscopy images of BEAS-2B cells captured in our study**

Figure S4. **Examples of automatically detected γH2AX binary objects and chromosomal regions in BEAS-2B cells**

Figure S5. **Maximum test balanced accuracies of cascade classifiers based on different numbers of top selected BEAS-2B phenotypic features and cell count**

Figure S6. **Chemical-wise classification accuracies of cascade classifiers based on the best BEAS-2B or A549 γH2AX feature and cell count**

Figure S7. **The F_2 values at lower concentrations for the four chemicals that cause major cell lost at the highest tested concentration**

Figure S8. **Microscopy images showing the anti-γH2AX staining patterns in BEAS-2B cells exposed to the indicated chemicals and concentrations for 16 hrs**

Figure S9. **Scatter plot showing mean cellular γH2AX intensity levels versus spatial cross-correlation of cellular DNA and γH2AX intensities in BEAS-2B cells**

Figure S10. **Microscopy images showing examples of DNA migration patterns obtained from the Comet assays for BEAS-2B cells exposed with diacetyl, nitrofurantoin, and *p*-phenylenediamine for 4 hrs**

Table S1. **List of candidate and selected chemicals**

Table S2. **Descriptions and references for chemical pulmonotoxicity annotations**

Table S3. **Phenotypic features used in our study**

Table S4. **DNA strand break measurements using the Comet assays**

Table S5. **Proposed lung adverse outcome pathways under development**

References

Supplementary Material 2. **Normalized phenotypic feature and cell viability values**

Supplementary Material 3. **Prediction performances of individual phenotypic features**

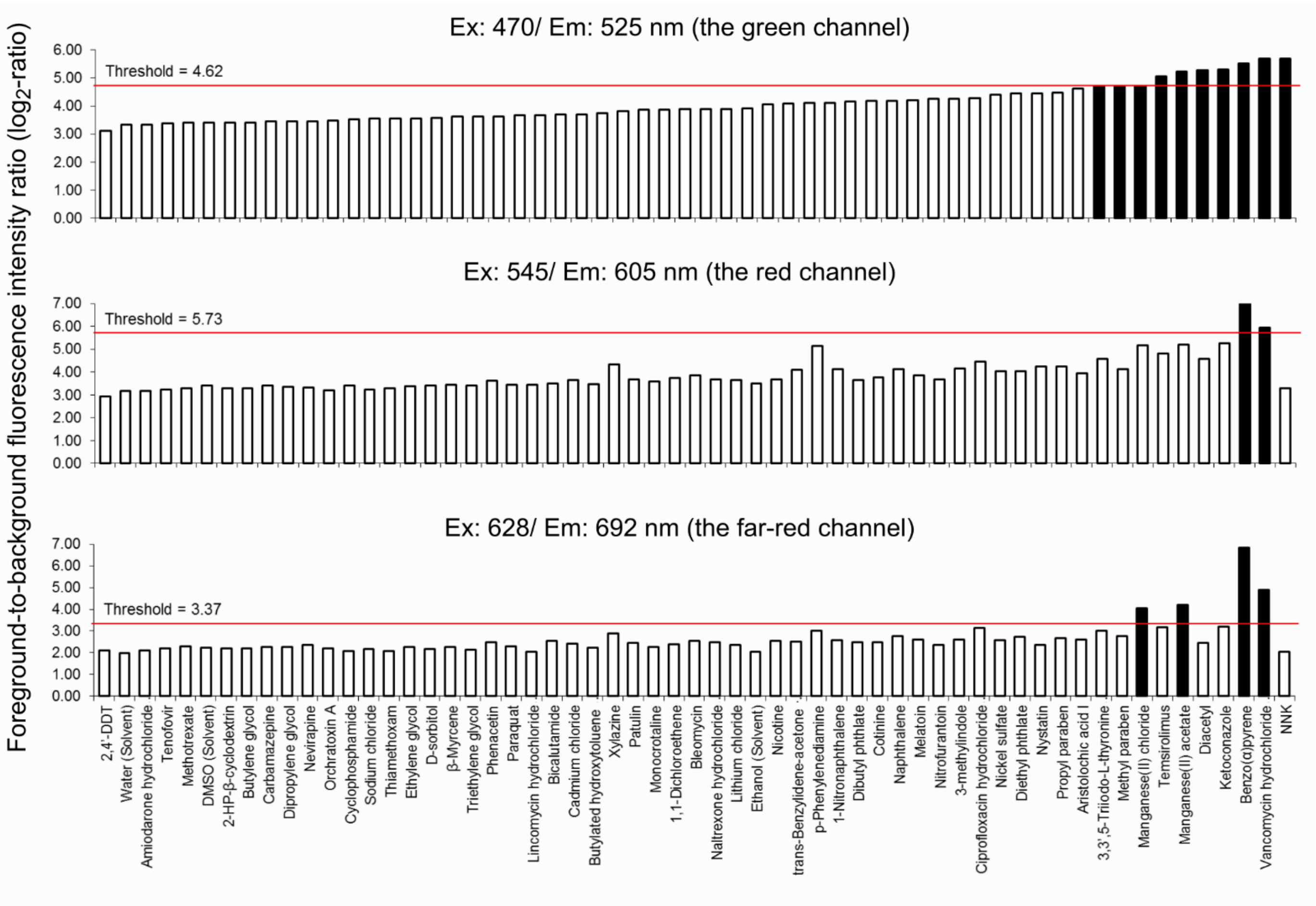

Figure S1. Detection of autofluorescent chemicals. Foreground-to-background fluorescence intensity ratios (FBRs) measured on BEAS-2B cells treated with the indicated chemicals or solvent controls for 16 hrs. The tested concentrations are provided in **Methods**. The FBRs were measured for the three indicated fluorescence channels (Ex = excitation wavelength, Em = emission wavelength) at 5000 ms, which is higher than the typical imaging exposure times that we used for these channels. The cells were only stained with Hoechst to allow automated quantification of FBRs. The maximum acceptable FBRs (red lines) were computed based on the positive control samples, and used to detect highly-autofluorescent chemicals (black bars) and choose the appropriate alternative fluorescent dyes for these chemicals (**Methods**). Four chemicals, namely benzo[α]pyrene, manganese(II) acetate, manganese(II) chloride, and vancomycin, were found to be highly autofluorescent in both the green and far-red channels, and thus are unsuitable for our imaging assay.

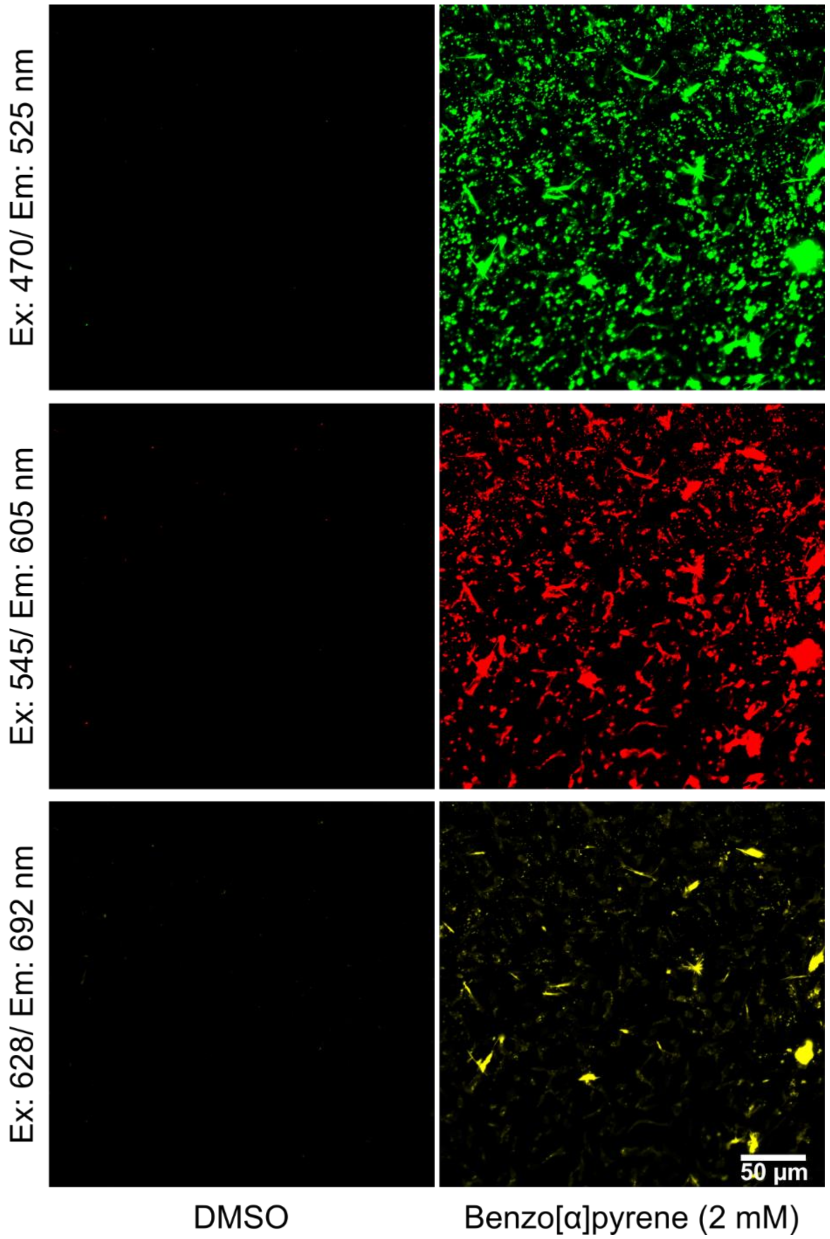

Figure S2. Benzo[α]pyrene is strongly autofluorescent in our three imaging channels. Microscopy images of BEAS-2B cells treated with 2 mM of benzo[α]pyrene or DMSO for 16 hrs, and imaged at the indicated fluorescence channels (scale bar = 50 µm). To allow visual comparisons, all the shown images have been scaled to the same intensity ranges.

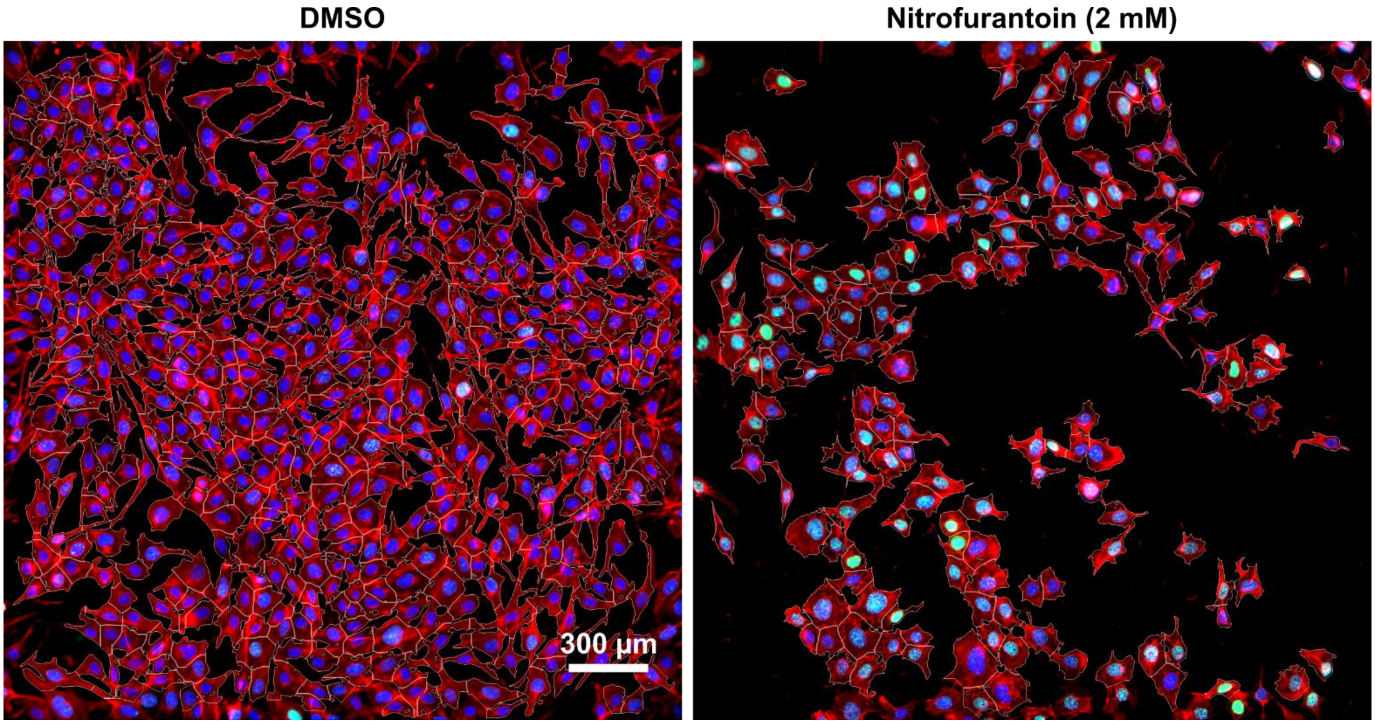

Figure S3. Examples of full-frame microscopy images of BEAS-2B cells captured in our study. Full-frame (uncropped) microscopy images of BEAS-2B cells treated with 2 mM of nitrofurantoin or DMSO for 16 hrs, and stained with the DNA (blue), γH2AX (green), actin (red) markers (white lines = automated cell segmentation boundaries, scale bar = 300 µm).

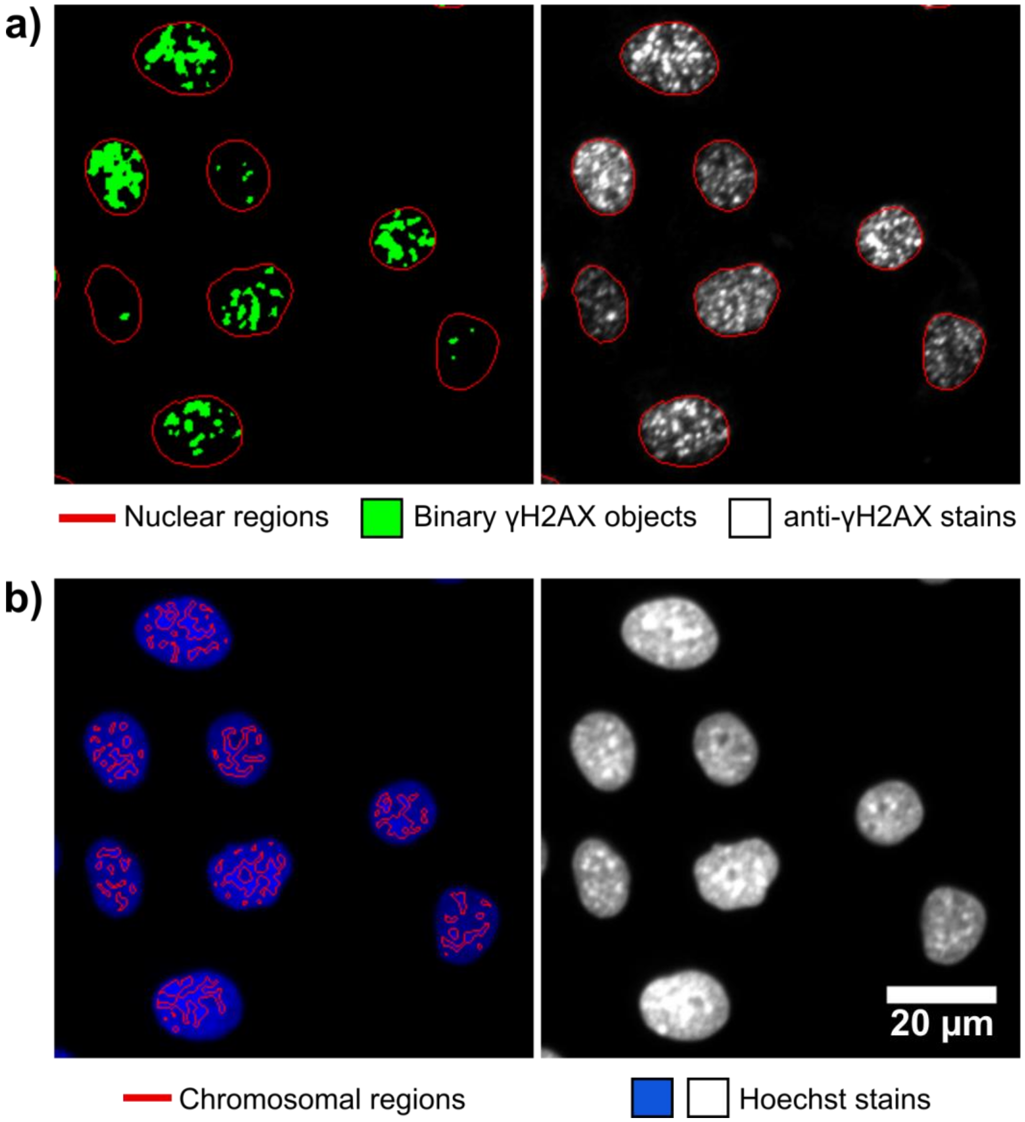

Figure S4. Examples of automatically detected γH2AX binary objects and chromosomal regions in BEAS-2B cells. Cropped microscopy images of BEAS-2B cells showing **a)** automatically detected γH2AX binary objects (green) and nuclear regions (red lines), and anti-γH2AX stains (white), and **b)** automatically detected chromosomal regions (red lines) and Hoechst stains (blue and white) on the same cells (scale bar = 20 µm).

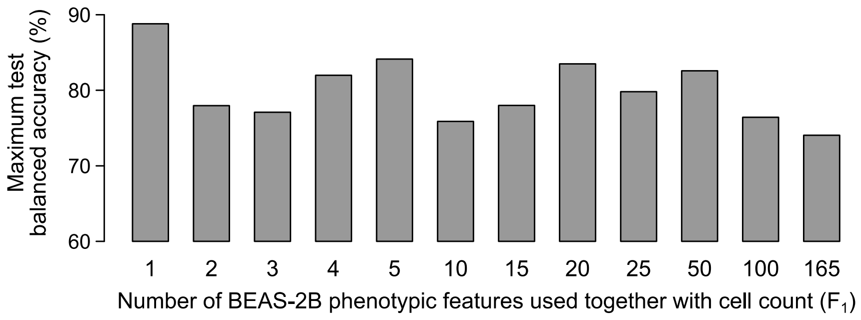

Figure S5. Maximum test balanced accuracies of cascade classifiers based on different numbers of top selected BEAS-2B phenotypic features and cell count. All values were estimated based on the reference chemicals using a 10-fold cross-validation procedure.

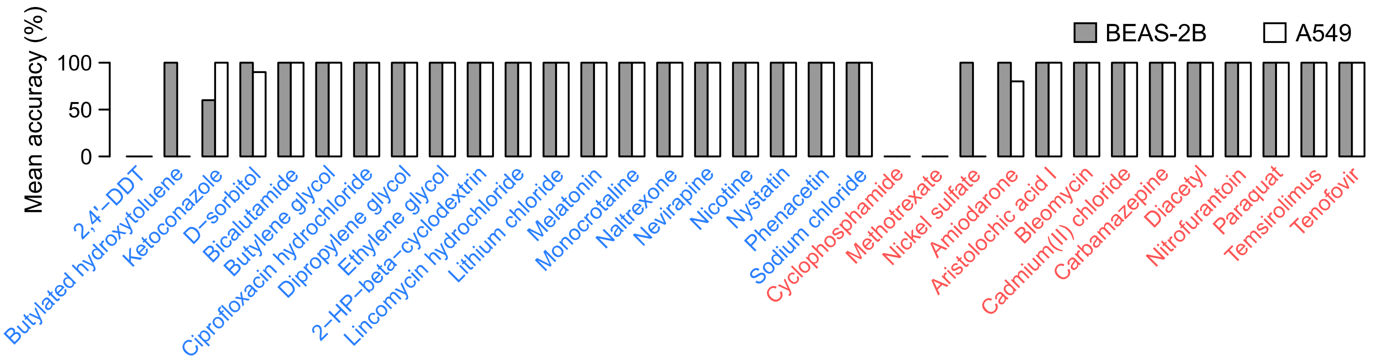

Figures S6. Chemical-wise classification accuracies of cascade classifiers based on the best BEAS-2B or A549 γH2AX feature and cell count. The values were extracted and averaged from the full 10-fold cross-validation results in classifying all the reference chemicals (dark gray = the best BEAS-2B γH2AX feature, white = the best A549 γH2AX feature).

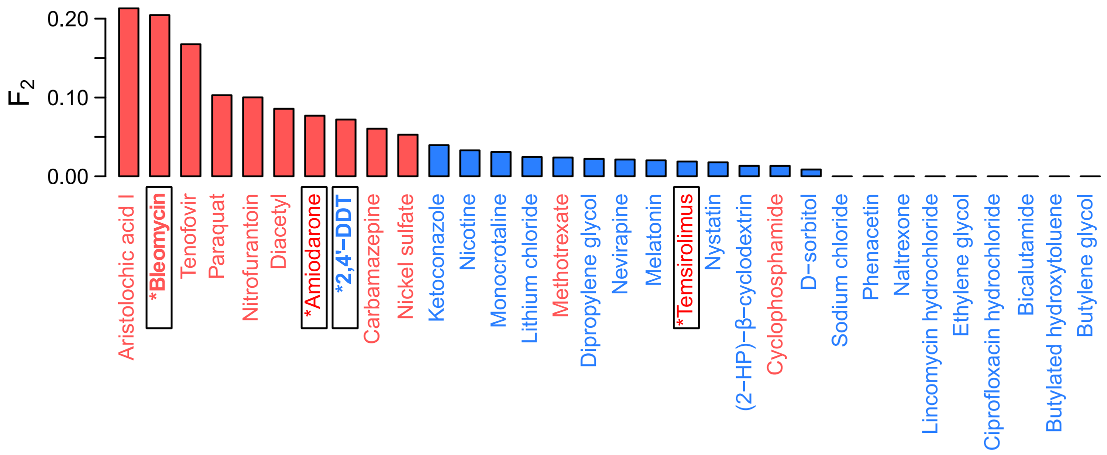

Figure S7. The F_2 values at lower concentrations for the four chemicals that cause major cell lost at the highest tested concentration. (* or boxes = the F_2 values for these chemicals were determined at the highest tested concentrations that induced <90% cell lost, please refer to the main text for the exact concentration values used; the F_2 values for all other chemicals were determined at the highest tested concentration; red or blue texts = chemicals labelled as pulmonotoxic or non-pulmonotoxic, respectively; red or blue bars = chemicals predicted to be "positive" or "negative", respectively.)

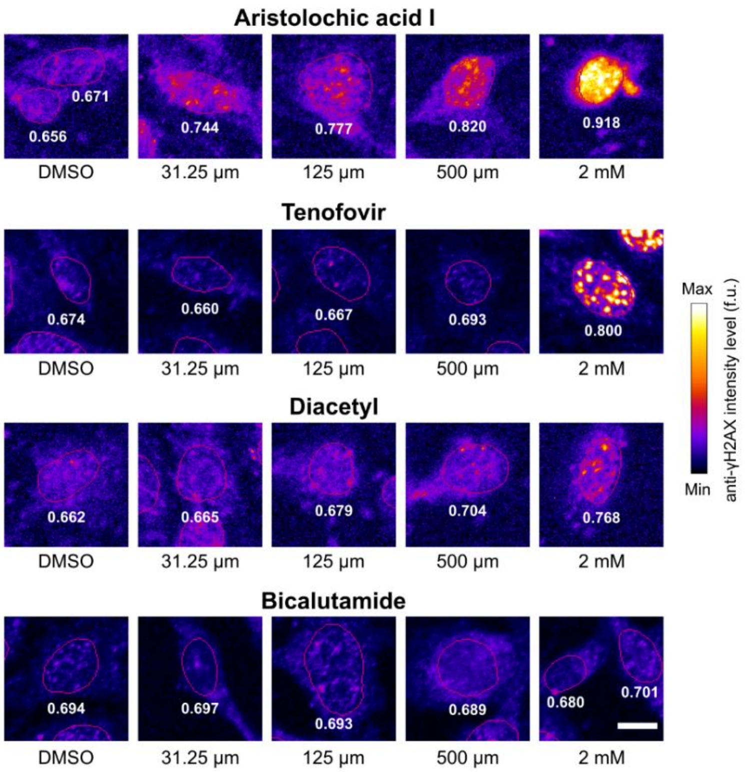

Figure S8. Microscopy images showing the anti-γH2AX staining patterns in BEAS-2B cells exposed to the indicated chemicals and concentrations for 16 hrs. (Scale bar = 30 μm, red lines = nuclear regions.)

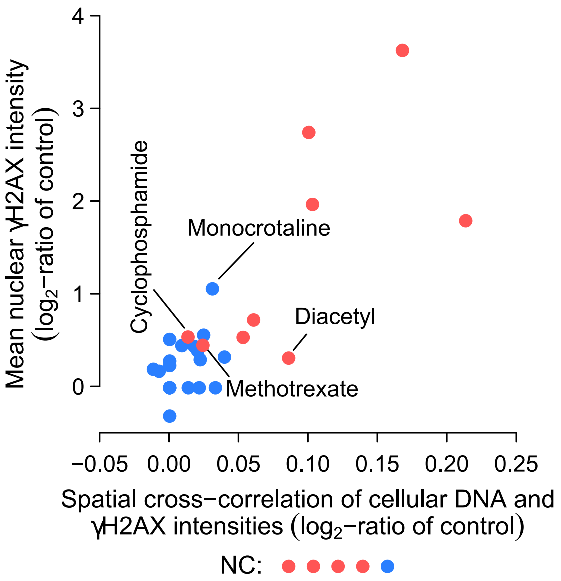

Figure S9. Scatter plot showing mean cellular γH2AX intensity levels versus spatial cross-correlation of cellular DNA and γH2AX intensities in BEAS-2B cells. (Red = pulmonotoxic reference chemicals, blue = non-pulmonotoxic reference chemicals.)

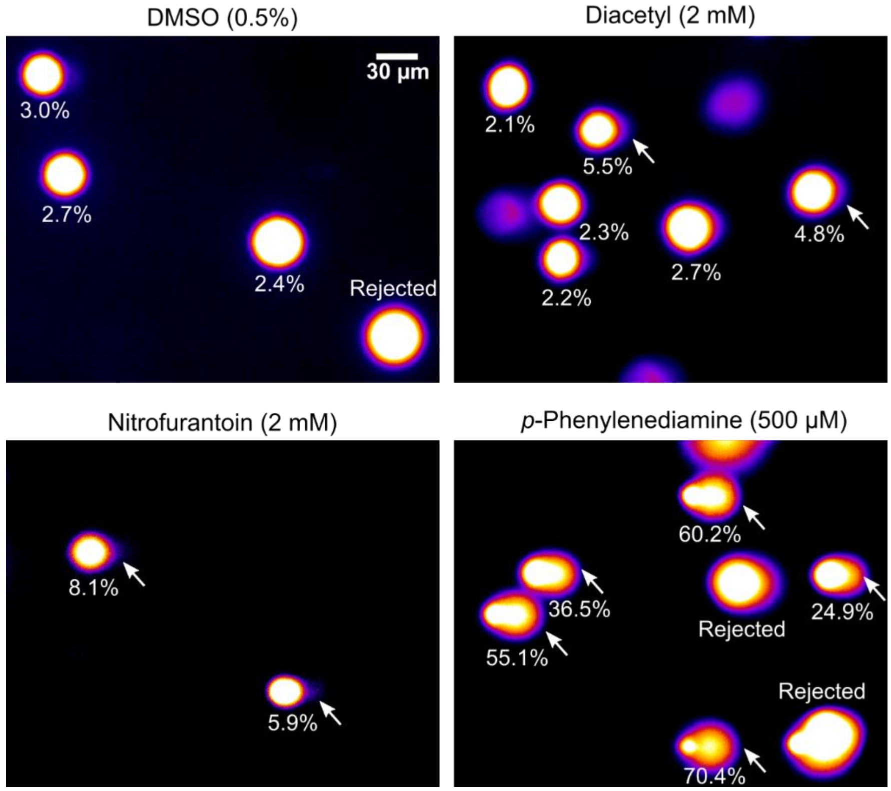

Figure S10. Microscopy images showing examples of DNA migration patterns obtained from the Comet assays for BEAS-2B cells exposed with diacetyl, nitrofurantoin, and *p*-phenylenediamine for 4 hrs. (Arrows = DNA migration patterns used for the quantifications, rejected = patterns rejected either by OpenComet or manual examinations.)

Table S1. List of candidate and selected chemicals

No.	Compound name	CAS number	Supplier	Catalog number	Solvent	Chemical quality control tests		
						Solubility	Autofluo-rescence	Imaging
**Pharmaceuticals**								
1	Amiodarone hydrochloride	19774-82-4	Cayman chemical	15213	DMSO	Passed	Passed	Passed
2	Bicalutamide	90357-06-5	Cayman chemical	14250	DMSO	Passed	Passed	Passed
3	Bleomycin sulfate	9041-93-4	Cayman chemical	13877	DMSO	Passed	Passed	Passed
4	Carbamazepine	298-46-4	Cayman chemical	15422	DMSO	Passed	Passed	Passed
5	Ciprofloxacin hydrochloride	93107-08-5	Cayman chemical	14286	Water	Passed	Passed	Passed
6	Cyclophosphamide	6055-19-2	Cayman chemical	13849	Water	Passed	Passed	Passed
7	2-Hydroxypropyl-β-cyclodextrin	128446-35-5	Cayman chemical	16169	DMSO	Passed	Passed	Passed
8	Ketoconazole	65277-42-1	Cayman chemical	15212	DMSO	Passed	Passed	Passed
9	Lincomycin hydrochloride	859-18-7	Sigma-Aldrich	62143	Water	Passed	Passed	Passed
10	Melatonin	73-31-4	Cayman chemical	14427	DMSO	Passed	Passed	Passed
11	Methotrexate	59-05-2	Sigma-Aldrich	PHR1396	DMSO	Passed	Passed	Passed
12	Naltrexone hydrochloride	16676-29-2	Cayman chemical	15520	Water	Passed	Passed	Passed
13	Nevirapine	129618-40-2	Sigma-Aldrich	SML0097	DMSO	Passed	Passed	Passed
14	Nitrofurantoin	67-20-9	Sigma-Aldrich	N7878	DMSO	Passed	Passed	Passed
15	Nystatin	1400-61-9	Sigma-Aldrich	N4014	DMSO	Passed	Passed	Passed
16	Phenacetin	62-44-2	Sigma-Aldrich	77440	DMSO	Passed	Passed	Passed
17	Temsirolimus	162635-04-3	Cayman chemical	11590	DMSO	Passed	Passed	Passed
18	Tenofovir	147127-20-6	Cayman chemical	13874	DMSO	Passed	Passed	Passed
19	Xylazine	7361-61-7	Cayman chemical	14113	DMSO	Passed	Passed	Passed
20	3,3',5-Triiodo-L-thyronine	6893-02-3	Sigma-Aldrich	T2877	DMSO	Passed	Passed	Failed
21	Vancomycin hydrochloride	1404-93-9	Sigma-Aldrich	V2002	Water	Passed	Failed	N/A
22	Barium sulfate	7727-43-7	Sigma-Aldrich	243353	Water	Failed	N/A	N/A
**Food ingredients or toxins**								
23	Aristolochic acid I	313-67-7	Sigma-Aldrich	A5512	DMSO	Passed	Passed	Passed
24	Diacetyl	431-03-8	Sigma-Aldrich	B85307	DMSO	Passed	Passed	Passed
	Butylated hydroxytoluene (BHT)	128-37-0	Sigma-Aldrich	B1378	EtOH	Passed	Passed	Passed
26	D-sorbitol	50-70-4	Sigma-Aldrich	240850	Water	Passed	Passed	Passed
27	Monocrotaline	315-22-0	Cayman chemical	16666	EtOH	Passed	Passed	Passed
28	Ochratoxin A	303-47-9	Cayman chemical	11439	DMSO	Passed	Passed	Passed
29	Patulin	149-29-1	Cayman chemical	11346	DMSO	Passed	Passed	Passed
30	Sodium chloride	7647-14-5	Sigma-Aldrich	S7653	Water	Passed	Passed	Passed
**Industrial chemicals**								
31	Cadmium chloride	10108-64-2	Sigma-Aldrich	202908	Water	Passed	Passed	Passed
32	1,1-Dichloroethene	75-35-4	Sigma-Aldrich	163023	DMSO	Passed	Passed	Passed
33	Ethylene glycol	107-21-1	Sigma-Aldrich	293237	Water	Passed	Passed	Passed
34	Lithium chloride	7447-41-8	Sigma-Aldrich	L4408	Water	Passed	Passed	Passed
35	Nickel(II) sulfate	10101-97-0	Sigma-Aldrich	227676	Water	Passed	Passed	Passed
36	Triethylene glycol	112-27-6	Sigma-Aldrich	T59455	Water	Passed	Passed	Passed
37	Manganese(II) acetate	6156-78-1	Sigma-Aldrich	63537	Water	Passed	Failed	N/A
38	Manganese(II) chloride	13446-34-9	Sigma-Aldrich	M3634	Water	Passed	Failed	N/A
39	Ferrocene	102-54-5	Sigma-Aldrich	F408	DMSO	Failed	N/A	N/A

No.	Compound name	CAS number	Supplier	Catalog number	Solvent	Chemical quality control tests		
						Solubility	Autofluo-rescence	Imaging
40	Gallium(III) oxide	12024-21-4	Sigma-Aldrich	215066	Water	Failed	N/A	N/A
41	Iron(III) oxide	1309-37-1	Sigma-Aldrich	310050	Water	Failed	N/A	N/A
**Consumer products**								
42	Butylene glycol (1,3-Butanediol)	107-88-0	Sigma-Aldrich	B84785	Water	Passed	Passed	Passed
43	*trans*-Benzylidene-acetone (Methyl styryl ketone)	1896-62-4	Sigma-Aldrich	241091	EtOH	Passed	Passed	Passed
44	Dibutyl phthalate	84-74-2	Sigma-Aldrich	524980	DMSO	Passed	Passed	Passed
45	Diethyl phthalate	84-66-2	Sigma-Aldrich	524972	DMSO	Passed	Passed	Passed
46	Dipropylene glycol	25265-71-8	Sigma-Aldrich	D215554	Water	Passed	Passed	Passed
47	3-Methylindole (Skatole)	83-34-1	Sigma-Aldrich	M51458	DMSO	Passed	Passed	Passed
48	β-Myrcene	123-35-3	Sigma-Aldrich	64643	DMSO	Passed	Passed	Passed
49	*p*-Phenylene-diamine	106-50-3	Sigma-Aldrich	P6001	Water	Passed	Passed	Passed
50	Propyl paraben	94-13-3	Sigma-Aldrich	P53357	DMSO	Passed	Passed	Passed
51	Methyl paraben	99-76-3	Sigma-Aldrich	H5501	DMSO	Passed	Passed	Passed
**Environmental agents**								
52	2,4'-DDT	789-02-6	Sigma-Aldrich	N12708	DMSO	Passed	Passed	Passed
53	Naphthalene	91-20-3	Sigma-Aldrich	147141	DMSO	Passed	Passed	Passed
54	Nicotine	54-11-5	Sigma-Aldrich	N3876	DMSO	Passed	Passed	Passed
55	Nicotine-derived nitrosamine ketone (NNK)	64091-91-4	Sigma-Aldrich	78013	Water	Passed	Passed	Passed
56	1-Nitro-naphthalene	86-57-7	Sigma-Aldrich	103594	EtOH	Passed	Passed	Passed
57	Paraquat	75365-73-0	Sigma-Aldrich	856177	Water	Passed	Passed	Passed
58	Thiamethoxam	153719-23-4	Sigma-Aldrich	37924	DMSO	Passed	Passed	Passed
59	Cotinine	486-56-6	Sigma-Aldrich	C5923	DMSO	Passed	Passed	Failed
60	Benzo[α]pyrene	50-32-8	Sigma-Aldrich	B1760	DMSO	Passed	Failed	N/A

Table S2. Descriptions and references for chemical pulmonotoxicity annotations

No.	Compound name	DailyMed	Human data	Animal data	Descriptions and references
**Pharmaceuticals**					
1	Amiodarone hydrochloride	Y	Pneumonitis and fibrosis (~4-9%)	Pneumonitis	In humans, amiodarone may cause pneumonitis and pulmonary fibrosis in ~4-9% patients [1]. In rats, amiodarone may cause pneumonitis [2].
2	Bleomycin sulfate	Y	Pneumonitis and fibrosis (~10%)	Pneumonitis and fibrosis	In humans, bleomycin may cause pneumonitis and pulmonary fibrosis in ~10% of the patients [3]. In dogs, hamsters, rats, and other animals, bleomycin may cause pneumonitis and pulmonary fibrosis [4].
3	Carba-mazepine	Y	Pneumonitis and pulmonary edema	No pulmonary toxicity found.	In humans, carbamazepine may cause pneumonitis and pulmonary edema [5–8], but we could not find information regarding their prevalence levels. In rats and dogs, acute, subacute and chronic studies did not found any pulmonary adverse effect [6,9].
4	Cyclo-phosphamide	Y	Pneumonitis and fibrosis	Pneumonitis, fibrosis, and tumors	In humans, cyclophosphamide may cause pneumonitis and pulmonary fibrosis [10,11]. In mice, cyclophosphamide may increase the occurrence of pneumonitis, pulmonary fibrosis, and lung tumors [11–13].
5	Methotrexate	Y	Pneumonitis and fibrosis (~2-33%)	Fibrosis	In humans, methotrexate may cause pneumonitis and pulmonary fibrosis [14,15]. The incidence of methotrexate-related pulmonary disease has been reported to be as low as 2-8% and as high as 33% [15]. In mice, methotrexate may increase the occurrence of pulmonary fibrosis [16,17].
6	Nitrofurantoin	Y	Pneumonitis and fibrosis	Respiratory distress and pulmonary edema (subcutaneous administration)	In humans, nitrofurantoin may cause pneumonitis and pulmonary fibrosis [18]. In rats, subcutaneous administration of nitrofurantoin may cause pulmonary edema, congestion, and haemorrhage [19]. However, another study of orally administered nitrofurantoin in rats and mice did not find major pulmonary effects [20].
7	Temsirolimus	Y	Interstitial lung disease (~7%)	No relevant pulmonary toxicity found.	In humans, temsirolimus may cause interstitial lung disease [21]. In a clinical study of east Asian patients, temsirolimus caused interstitial lung disease in ~7% of the patients [22]. In animals, temsirolimus may increase alveolar macrophages in rats, but not in mice or monkeys [23,24]. The appearance and aetiology of the changes in the lung seen in rats are distinct from interstitial pneumonitis (observed in clinical studies with temsirolimus) and a relationship between the two conditions is considered unlikely [24].
8	Tenofovir	Y	Pneumonia (~2-5%)	No pulmonary toxicity found.	In humans, tenofovir may cause pneumonia or bronchitis in ~2-5% of the patients [25,26]. In rats and dogs, preclinical assessment of tenofovir did not find major pulmonary toxicity [26].
9	Bicalutamide	Y	Rare pneumonitis and fibrosis (<1%).	No pulmonary toxicity found.	In humans, rare pneumonitis and pulmonary fibrosis were found in <1% of the patients taking bicalutamide [27,28]. In rats, mice, dogs and other animals, no pulmonary toxicity has been reported [29,30].

No.	Compound name	DailyMed	Human data	Animal data	Descriptions and references
10	Ciprofloxacin hydrochloride	Y	Rare pulmonary edema (<1%).	No pulmonary toxicity found.	In humans, rare dyspnea, epistaxis, pulmonary edema, or other pulmonary effects were found in <1% of the patients [31,32]. In animals, no pulmonary toxicity has been reported [33,34].
11	2-Hydroxypropyl-β-cyclodextrin	N	No pulmonary toxicity found.	Pneumonitis	In humans, no pulmonary toxicity was reported in several clinical studies of orally or intravenously delivered 2-hydroxypropyl-β-cyclodextrin [35]. In rats, reversible macrophage infiltration in the lung, with some associated alveolitis haemorrhage and atelectasis, was observed [35].
12	Ketoconazole	Y	No pulmonary toxicity found.	No pulmonary toxicity found.	In humans, the major adverse effects of ketoconazole observed in clinical trials or post-marketing surveillances are hepatotoxicity and adrenal insufficiency [36–38]. In animals, pathological changes in liver, kidney, adrenal and ovaries were observed in rats and dogs for studies up to 12 months [36]. No major pulmonary effect was reported for both humans and animals [36–38].
13	Lincomycin hydrochloride	Y	No pulmonary toxicity found.	No pulmonary toxicity found.	In humans, no pulmonary toxicity was reported [39]. In rats and dogs, no pulmonary toxicity was found in orally delivered lincomycin [40].
14	Melatonin	Y	No pulmonary toxicity found.	No pulmonary toxicity found.	In humans and animals, no major chemical-induced pulmonary toxicity was reported [41–43].
15	Naltrexone hydrochloride	Y	No pulmonary toxicity found.	No pulmonary toxicity found.	In humans, rare pulmonary effects were found in <1% of patients [44]. In rats, dogs, and monkeys, no major chemical-induced pulmonary toxicity was reported [45].
16	Nevirapine	Y	No pulmonary toxicity found.	No pulmonary toxicity found.	In humans, rats, and dogs, no major chemical-induced pulmonary toxicity was reported [46,47].
17	Nystatin	Y	No pulmonary toxicity found.	No pulmonary toxicity found.	In humans and mice, no major chemical-induced pulmonary toxicity was reported [48–51].
18	Phenacetin	Y	No pulmonary toxicity found.	No pulmonary toxicity found.	In humans and animals, no major chemical-induced pulmonary toxicity was reported [52,53].
19	Xylazine	N	May cause pulmonary congestion and edema, but most patients fully recovered. So, the direct pulmonary effects are unclear.	Pulmonary edema.	In humans, accidental injection or ingestion of xylazine often leads to central nervous system depression and respiratory distress; but in most cases, the patients fully recovered [54]. However, in a case of fatal abuse, autopsy of the body found congested and edematous lungs [55]. Therefore, it is unclear if xylazine is directly toxic to lung cells, or the pulmonary effects are secondary to the CNS depression. In rats, xylazine may cause pulmonary edema [56].
**Food ingredients or toxins**					
20	Aristolochic acid I	N	DNA-adducts in lungs	DNA-adducts in lungs and tumor	The major target organ of aristolochic acids is the kidney. However, in both humans and animals, DNA adducts may be found in the lungs [57,58]. In mice, aristolochic acids may increase the occurrence of lung and other tumors [57].

No.	Compound name	DailyMed	Human data	Animal data	Descriptions and references
21	Diacetyl	N	Bronchiolitis obliterans (epidemiological studies only)	Bronchitis and fibrosis	In humans, several epidemiological studies found that workers exposed to diacetyl are more likely to develop bronchiolitis obliterans [59,60]. In rats and mice, acute and sub chronic studies found that diacetyl can damage lung cells [61,62].
22	Butylated hydroxytoluene (BHT)	N	No pulmonary toxicity found.	Recoverable pulmonary inflammation and necrosis	In humans, ingestion of BHT may not induce major pulmonary toxicity [63–65]. In mice, BHT may produce early but recoverable pulmonary inflammation and injury, including necrosis of alveolar cells [65].
23	D-sorbitol	Y	No pulmonary toxicity found.	No pulmonary toxicity found.	In humans and rats, no pulmonary toxicity was reported for dietary intake of sorbitol [66].
24	Monocrotaline	N	No pulmonary toxicity found.	Pulmonary edema and hypertension.	Monocrotaline is a pyrrolizidine alkaloid (PA) derived from *Crotalaria spectabilis*. In humans, the toxic effects of PAs are principally on the liver, including haemorrhagic necrosis and/or veno-occlusive disease [67,68]. There was no substantiated report of primary pulmonary hypertension resulting from PA poisoning in humans [67,68]. In rats, monocrotaline may injure pulmonary endothelial cell and cause pulmonary hypertension and edema [67,69,70].
25	Sodium chloride	N	No pulmonary toxicity found.	No pulmonary toxicity found.	In humans and animals, no major chemical-induced pulmonary toxicity was reported [71]. Intravenous administration of sodium chloride may cause pulmonary edema in humans, but the effect is likely due to solute overload [71].
26	Ochratoxin A	N	No relevant study found.	No direct pulmonary toxicity found.	In humans, no relevant study was found regarding the pulmonary effects of ochratoxin A [72]. Most of the human studies focused on the kidney effects of ochratoxin A [72,73]. In rats, no significant chemical-induced pulmonary toxicity was reported in subacute or subchronic studies, and the major target was kidney tubular cells [72,74]. However, in a 2-year study, the lungs were the most common metastatic site for renal carcinoma cells [74]. Interestingly, in rats, lungs were found to have the highest distribution of ingested or intravenous-injected ochratoxin A [72].
27	Patulin	N	No relevant study found.	Atelectasis and pulmonary edema.	In humans, no relevant study was found regarding the pulmonary effects of patulin [75]. In rats and mice, patulin may cause atelectasis, alveolar septal congestion, and/or limited intra-alveolar haemorrhage in the lungs [76]. In dogs, patulin may also cause pulmonary haemorrhage and edema [77].
**Industrial chemicals**					
28	Cadmium chloride	N	Bronchiolitis, pneumonitis and pulmonary edema	Pneumonitis and fibrosis	In humans and animals, inhalation of cadmium chloride may cause bronchiolitis, pneumonitis and pulmonary edema [78].
29	Nickel (II) sulfate	N	Obstructive lung disease and fibrosis	Pneumonitis and fibrosis	In humans, inhalation exposure of nickel may cause obstructive pulmonary disease, fibrosis, and edema [79]. In rats and mice, inhalation of nickel may cause pneumonitis and fibrosis [79,80].

No.	Compound name	DailyMed	Human data	Animal data	Descriptions and references
30	Ethylene glycol	N	Rare pulmonary edema, which are secondary to cardiac failure.	No pulmonary toxicity found.	In humans, inhalation of ethylene glycol may cause tolerable nose and throat irritations [81]. Major respiratory effects, such as pulmonary edema, are relatively rare and usually occur concomitantly with cardiovascular changes. Thus they are likely to be secondary to cardiac failure [81]. In rodents, no lung toxicity was observed in both acute and chronic oral studies [81].
31	Lithium chloride	N	No pulmonary toxicity found.	No pulmonary toxicity found.	In humans and animals, no major chemical-induced pulmonary toxicity was reported [82–84].
32	1,1-Dichloroethene	N	No relevant study found.	Pulmonary edema	In humans, no relevant study was found regarding the respiratory effects of 1,1-dichloroethene [85,86]. In rodents, inhalation of 1,1-dichloroethene may cause pulmonary edema [85].
33	Triethylene glycol	N	No relevant study found.	Histological changes in the lungs.	In humans, no relevant study was found regarding the direct respiratory effects of triethylene glycol [87]. In rats and rabbits, acute treatments of triethylene glycol may cause histological changes in the lungs [87].
**Personal-care or consumer products**					
34	Butylene glycol (1,3-butanediol)	N	No pulmonary toxicity found.	No pulmonary toxicity found.	In humans, rats, and dogs, no pulmonary toxicity was reported in several studies of dietary intake of 1,3-butanediol [88].
35	Dipropylene glycol	N	No pulmonary toxicity found for dermal exposure, or expected for oral exposure	No pulmonary toxicity found.	In humans, no pulmonary effect was reported for dermal exposure of dipropylene glycol [89]. In rats and mice, no pulmonary effect was reported for oral exposure of dipropylene glycol [90]. The metabolism of mono-, di-, and tri-propylene glycols share common pathways and a consistent profile of toxicity is observed [90]. Thus, read-across may be used to describe potential hazards for specific oligomers with data gaps [90]. In both humans and rats, no pulmonary effect was reported for oral exposure of monopropylene glycol [90,91]. Thus, dipropylene glycol is expected to have similar effects with oral exposures.
36	*trans*-Benzylidene-acetone (Methyl styryl ketone)	N	No relevant study found.	No pulmonary toxicity found.	No record was found in NLM Hazardous Substances Data Bank. In rats, there was an increased incidence of goblet cell hyperplasia of the respiratory epithelium of the nose, but no other major chemical-induced pulmonary toxicity was found [92]. In mice, no major chemical-induced pulmonary toxicity was found [92].
37	Dibutyl phthalate	N	No relevant study found.	No pulmonary toxicity found.	In humans, no relevant study was found regarding the pulmonary toxicity of dibutyl phthalate [93]. In rats and mice, no significant chemical-induced pulmonary toxicity was reported [94]. However, dibutyl phthalate may reduce the lung concentration of cytochrome P-450 [95,96]. The main targets of dibutyl phthalate in rats may be the liver and testes [94].
38	Diethyl phthalate	N	No relevant study found.	No pulmonary toxicity found.	In humans, no relevant study was found regarding the pulmonary toxicity of diethyl phthalate [97,98]. In animals, no significant chemical-induced pulmonary toxicity was reported [97].

No.	Compound name	DailyMed	Human data	Animal data	Descriptions and references
39	3-Methylindole (Skatole)	N	No relevant study found.	Pulmonary edema.	In humans, no relevant study was found regarding the pulmonary effects of 3-methylindole [99]. In cattle, sheep, and goats, 3-methylindole may cause pulmonary edema and interstitial emphysema [100–102].
40	Methyl paraben	N	No relevant study found.	Ciliotoxicity, which impairs the ciliary motion of mucous membrane cilia. Direct effects to lung cells are unclear.	In humans, no relevant study was found regarding the pulmonary effects of methyl paraben [103]. In rats, methyl paraben is mildly ciliotoxic when inhaled. When administered orally to rats, congested lungs were observed, but the results were not reproducible in subsequent experiments [114].
41	β-Myrcene	N	No relevant study found.	No pulmonary toxicity found	In humans, no relevant study was found regarding the pulmonary effects of β-myrcene [104]. In rats and mice, no significant chemical-induced pulmonary toxicity was reported [104,105].
42	*p*-Phenylene-diamine	N	Obstructive lung disease and pulmonary edema, but they may be due to rhabdomyolysis. Direct effects to lungs are unclear.	No pulmonary toxicity found.	In humans, pulmonary obstruction is a major cause of death and complication in p-phenylenediamine poisoning [106–108]. However, the effects may be secondary to rhabdomyolysis, and the direct pulmonary effects of p-phenylenediamine is unclear. In animals, p-phenylenediamine may cause skeletal muscle damage and rhabdomyolysis, but no major effect on the lungs have been reported [109–111]. In mice, alveolar bronchiolar adenomas was found in a study, but the finding was deemed equivocal [110,112].
43	Propyl paraben	N	No relevant study found.	No pulmonary toxicity found.	In humans, no relevant study was found regarding the pulmonary effects of propyl paraben [103]. In animals, no significant chemical-induced pulmonary toxicity was reported [103].
**Environmental agents**					
44	Paraquat	N	Pulmonary edema and Fibrosis	Fibrosis	In humans, paraquat may cause alveolar edema and pulmonary fibrosis [113,114]. In rats, guinea pigs, and monkeys, paraquat may cause lung fibrosis [115].
45	2,4’-DDT	N	No pulmonary toxicity found.	No pulmonary toxicity found.	In humans, no pulmonary toxicity was reported in an inhalation study of DDT [116]. In a historical cohort study, no evidence for a link between occupational exposure to DDT and mortality from lung or other type of cancers was found [117]. The effects of ingested DDT on the respiratory system are likely to be secondary to the effects on the nervous system [118]. In rats and mice, oral exposure to DDT did not cause pulmonary toxicity [116].
46	Nicotine	Y	No direct pulmonary toxicity found.	No pulmonary toxicity found.	In humans, no major pulmonary toxicity was reported for clinical trials of nicotine replacement therapy [119,120]. One of the most common causes of death from nicotine poisoning is respiratory failure due to peripheral neuromuscular blockade and cardiovascular arrest [121]. In rat, chronic inhalation exposure to nicotine does not increase the occurrence of lung tumor [122].

No.	Compound name	DailyMed	Human data	Animal data	Descriptions and references
47	1-Nitro-naphthalene	N	No relevant study found.	Non-ciliated bronchiolar cell necrosis	In humans, no relevant study was found regarding the pulmonary effects of 1-nitronaphthalene [123]. In rats, acute intraperitoneal injection of 1-nitronaphthalene may cause respiratory distress, non-ciliated bronchiolar cell necrosis, and Interstitial pneumonitis and edema [124,125].
48	Naphthalene	N	No relevant study found. Epidemiological studies are not conclusive.	Lung tumor and pulmonary fibrosis.	In humans, no relevant study was found regarding the pulmonary toxicity of inhaled naphthalene [126]. Epidemiological studies are not conclusive, but naphthalene is anticipated to be a human carcinogen [127]. No significant chemical-induced pulmonary toxicity was found in cases of naphthalene ingestion [126,128]. In mice, chronic inhalation studies found increased incidences of neoplastic and non-neoplastic lesions in the lungs [126]. No significant chemical-induced pulmonary toxicity was found in rats and mice for orally administered naphthalene [126].
49	Nicotine-derived nitrosamine ketone (NNK)	N	No relevant study found.	Lung tumor.	In humans, no relevant study was found regarding the pulmonary effects of NNK [129,130], and no epidemiological study could be identified that evaluated the relationship between human cancer and exposure specifically to NNK [129]. However, NNK is anticipated to be a human carcinogen [129]. In rodents, NNK may induce lung adenomas independent of the route of administration [129,130].
50	Thiamethoxam	N	No relevant study found.	No pulmonary toxicity found.	In humans, no relevant study was found regarding the respiratory effects thiamethoxam [131]. In rats and mice, no significant chemical-induced pulmonary toxicity was reported [131]. Effects on liver were observed [131].

Table S3. Phenotypic features used in our study

A feature name has three fields separated by colons (e.g., “total_intensity:Actin:cell_region”). The first field provides the name of the measurement (e.g., “total_intensity”). The second field provides the fluorescence markers used to measure the feature (e.g., “Actin”). For all morphology features, this field is labelled as “mask”, because the features were measured from binary masks representing different subcellular regions. The third field provides the subcellular regions where the feature was measured (e.g., “cell_region”).

Feature names	Types
area:mask:cell_region	Morphology
area:mask:dna_region	Morphology
perimeter:mask:cell_region	Morphology
perimeter:mask:dna_region	Morphology
roundness:mask:cell_region	Morphology
roundness:mask:dna_region	Morphology
aspect_ratio:mask:cell_region	Morphology
aspect_ratio:mask:dna_region	Morphology
obj_number:mask:DNA_object	Morphology
obj_mean_total_area:mask:DNA_object	Morphology
obj_stddev_total_area:mask:DNA_object	Morphology
obj_number:mask:gH2AX_object	Morphology
obj_mean_total_area:mask:gH2AX_object	Morphology
obj_stddev_total_area:mask:gH2AX_object	Morphology
obj_number:mask:Actin_object	Morphology
obj_mean_total_area:mask:Actin_object	Morphology
obj_stddev_total_area:mask:Actin_object	Morphology
total_intensity:gH2AX:cell_region	Intensity
total_intensity:Actin:cell_region	Intensity
mean_intensity:gH2AX:cell_region	Intensity
mean_intensity:Actin:cell_region	Intensity
cv_intensity:gH2AX:cell_region	Intensity
cv_intensity:Actin:cell_region	Intensity
total_intensity:DNA:dna_region	Intensity
total_intensity:DNA:dna_chromosome	Intensity
mean_intensity:DNA:dna_region	Intensity
mean_intensity:DNA:dna_chromosome	Intensity
cv_intensity:DNA:dna_region	Intensity
cv_intensity:DNA:dna_chromosome	Intensity
fraction_obj_intensity:DNA:dna_region-DNA_object	Intensity ratio
fraction_obj_intensity:DNA:dna_chromosome-DNA_object	Intensity ratio
fraction_obj_intensity:DNA:nondna_region-DNA_object	Intensity ratio
total_intensity:gH2AX:dna_region	Intensity
total_intensity:gH2AX:dna_chromosome	Intensity
mean_intensity:gH2AX:dna_region	Intensity
mean_intensity:gH2AX:dna_chromosome	Intensity

cv_intensity:gH2AX:dna_region	Intensity
cv_intensity:gH2AX:dna_chromosome	Intensity
total_intensity_ratio:gH2AX-gH2AX:dna_region-cell_region	Intensity ratio
total_intensity_ratio:gH2AX-gH2AX:dna_chromosome-cell_region	Intensity ratio
total_intensity_ratio:gH2AX-gH2AX:dna_chromosome-dna_region	Intensity ratio
fraction_obj_intensity:gH2AX:dna_region-gH2AX_object	Intensity ratio
fraction_obj_intensity:gH2AX:dna_chromosome-gH2AX_object	Intensity ratio
total_intensity:Actin:dna_region	Intensity
total_intensity:Actin:dna_chromosome	Intensity
total_intensity:Actin:nondna_region	Intensity
total_intensity:Actin:nondna_outer	Intensity
total_intensity:Actin:nondna_inner	Intensity
total_intensity:Actin:nondna_peridna	Intensity
mean_intensity:Actin:dna_region	Intensity
mean_intensity:Actin:dna_chromosome	Intensity
mean_intensity:Actin:nondna_region	Intensity
mean_intensity:Actin:nondna_outer	Intensity
mean_intensity:Actin:nondna_inner	Intensity
mean_intensity:Actin:nondna_peridna	Intensity
cv_intensity:Actin:dna_region	Intensity
cv_intensity:Actin:dna_chromosome	Intensity
cv_intensity:Actin:nondna_region	Intensity
cv_intensity:Actin:nondna_outer	Intensity
cv_intensity:Actin:nondna_inner	Intensity
cv_intensity:Actin:nondna_peridna	Intensity
total_intensity_ratio:Actin-Actin:dna_region-cell_region	Intensity ratio
total_intensity_ratio:Actin-Actin:dna_chromosome-cell_region	Intensity ratio
total_intensity_ratio:Actin-Actin:dna_chromosome-dna_region	Intensity ratio
total_intensity_ratio:Actin-Actin:nondna_outer-cell_region	Intensity ratio
total_intensity_ratio:Actin-Actin:nondna_inner-cell_region	Intensity ratio
total_intensity_ratio:Actin-Actin:nondna_peridna-cell_region	Intensity ratio
fraction_obj_intensity:Actin:dna_region-Actin_object	Intensity ratio
fraction_obj_intensity:Actin:dna_chromosome-Actin_object	Intensity ratio
fraction_obj_intensity:Actin:nondna_region-Actin_object	Intensity ratio
fraction_obj_intensity:Actin:nondna_outer-Actin_object	Intensity ratio
fraction_obj_intensity:Actin:nondna_inner-Actin_object	Intensity ratio
fraction_obj_intensity:Actin:nondna_peridna-Actin_object	Intensity ratio
total_intensity_ratio:gH2AX-Actin:cell_region-cell_region	Intensity ratio
total_intensity_ratio:gH2AX-DNA:cell_region-cell_region	Intensity ratio
total_intensity_ratio:DNA-Actin:cell_region-cell_region	Intensity ratio
total_intensity_ratio:gH2AX-Actin:dna_region-dna_region	Intensity ratio
total_intensity_ratio:gH2AX-DNA:dna_region-dna_region	Intensity ratio
total_intensity_ratio:DNA-Actin:dna_region-dna_region	Intensity ratio

total_intensity_ratio:gH2AX-Actin:dna_chromosome-dna_chromosome	Intensity ratio
total_intensity_ratio:gH2AX-DNA:dna_chromosome-dna_chromosome	Intensity ratio
total_intensity_ratio:DNA-Actin:dna_chromosome-dna_chromosome	Intensity ratio
ccorr_normed:DNA-gH2AX:cell_region	Correlation
ccoeff_normed:DNA-gH2AX:cell_region	Correlation
ccorr_normed:DNA-Actin:cell_region	Correlation
ccoeff_normed:DNA-Actin:cell_region	Correlation
ccorr_normed:gH2AX-Actin:cell_region	Correlation
ccoeff_normed:gH2AX-Actin:cell_region	Correlation
ccorr_normed:DNA-gH2AX:dna_region	Correlation
ccoeff_normed:DNA-gH2AX:dna_region	Correlation
ccorr_normed:DNA-Actin:dna_region	Correlation
ccoeff_normed:DNA-Actin:dna_region	Correlation
ccorr_normed:gH2AX-Actin:dna_region	Correlation
ccoeff_normed:gH2AX-Actin:dna_region	Correlation
ccorr_normed:DNA-gH2AX:dna_chromosome	Correlation
ccoeff_normed:DNA-gH2AX:dna_chromosome	Correlation
ccorr_normed:DNA-Actin:dna_chromosome	Correlation
ccoeff_normed:DNA-Actin:dna_chromosome	Correlation
ccorr_normed:gH2AX-Actin:dna_chromosome	Correlation
ccoeff_normed:gH2AX-Actin:dna_chromosome	Correlation
glcm_asm_mean:Actin:cell_region	Texture
glcm_contrast_mean:Actin:cell_region	Texture
glcm_corr_mean:Actin:cell_region	Texture
glcm_var_mean:Actin:cell_region	Texture
glcm_idm_mean:Actin:cell_region	Texture
glcm_sum_ave_mean:Actin:cell_region	Texture
glcm_sum_var_mean:Actin:cell_region	Texture
glcm_sum_ent_mean:Actin:cell_region	Texture
glcm_ent_mean:Actin:cell_region	Texture
glcm_diff_var_mean:Actin:cell_region	Texture
glcm_diff_ent_mean:Actin:cell_region	Texture
glcm_info_corr1_mean:Actin:cell_region	Texture
glcm_info_corr2_mean:Actin:cell_region	Texture
glcm_asm_mean:Actin:dna_region	Texture
glcm_contrast_mean:Actin:dna_region	Texture
glcm_corr_mean:Actin:dna_region	Texture
glcm_var_mean:Actin:dna_region	Texture
glcm_idm_mean:Actin:dna_region	Texture
glcm_sum_ave_mean:Actin:dna_region	Texture
glcm_sum_var_mean:Actin:dna_region	Texture
glcm_sum_ent_mean:Actin:dna_region	Texture
glcm_ent_mean:Actin:dna_region	Texture

glcm_diff_var_mean:Actin:dna_region	Texture
glcm_diff_ent_mean:Actin:dna_region	Texture
glcm_info_corr1_mean:Actin:dna_region	Texture
glcm_info_corr2_mean:Actin:dna_region	Texture
glcm_asm_mean:Actin:nondna_region	Texture
glcm_contrast_mean:Actin:nondna_region	Texture
glcm_corr_mean:Actin:nondna_region	Texture
glcm_var_mean:Actin:nondna_region	Texture
glcm_idm_mean:Actin:nondna_region	Texture
glcm_sum_ave_mean:Actin:nondna_region	Texture
glcm_sum_var_mean:Actin:nondna_region	Texture
glcm_sum_ent_mean:Actin:nondna_region	Texture
glcm_ent_mean:Actin:nondna_region	Texture
glcm_diff_var_mean:Actin:nondna_region	Texture
glcm_diff_ent_mean:Actin:nondna_region	Texture
glcm_info_corr1_mean:Actin:nondna_region	Texture
glcm_info_corr2_mean:Actin:nondna_region	Texture
glcm_asm_mean:DNA:dna_region	Texture
glcm_contrast_mean:DNA:dna_region	Texture
glcm_corr_mean:DNA:dna_region	Texture
glcm_var_mean:DNA:dna_region	Texture
glcm_idm_mean:DNA:dna_region	Texture
glcm_sum_ave_mean:DNA:dna_region	Texture
glcm_sum_var_mean:DNA:dna_region	Texture
glcm_sum_ent_mean:DNA:dna_region	Texture
glcm_ent_mean:DNA:dna_region	Texture
glcm_diff_var_mean:DNA:dna_region	Texture
glcm_diff_ent_mean:DNA:dna_region	Texture
glcm_info_corr1_mean:DNA:dna_region	Texture
glcm_info_corr2_mean:DNA:dna_region	Texture
glcm_asm_mean:gH2AX:dna_region	Texture
glcm_contrast_mean:gH2AX:dna_region	Texture
glcm_corr_mean:gH2AX:dna_region	Texture
glcm_var_mean:gH2AX:dna_region	Texture
glcm_idm_mean:gH2AX:dna_region	Texture
glcm_sum_ave_mean:gH2AX:dna_region	Texture
glcm_sum_var_mean:gH2AX:dna_region	Texture
glcm_sum_ent_mean:gH2AX:dna_region	Texture
glcm_ent_mean:gH2AX:dna_region	Texture
glcm_diff_var_mean:gH2AX:dna_region	Texture
glcm_diff_ent_mean:gH2AX:dna_region	Texture
glcm_info_corr1_mean:gH2AX:dna_region	Texture
glcm_info_corr2_mean:gH2AX:dna_region	Texture

Table S4. DNA strand break measurements using a Comet assay

* red = significant DNA strand breaks were observed

Chemical name	Chemical exposure time (hrs)	Median Percentage of Tail DNA (%)		Change in Median Percentage of Tail DNA (log2-ratio)			P-value	
		Mean of chemical replicates	Mean of solvent replicates	Lower 95% confidence limit	Mean	Upper 95% confidence limit	Raw	FDR-adjusted
Bleomycin	4	50.84	3.35	3.61	3.92	4.24	0.000	0.000
p-Phenylenediamine	4	57.01	3.16	3.18	4.17	5.17	0.001	0.006
Diacetyl	4	4.69	2.68	0.34	0.81	1.27	0.005	0.020
Nitrofurantoin	4	5.83	3.30	0.20	0.82	1.44	0.022	0.069
Nickel sulfate	4	3.99	2.89	0.03	0.46	0.89	0.042	0.114
Bicalutamide	4	4.60	3.14	-0.07	0.55	1.18	0.069	0.165
Temosirolimus	4	4.75	2.88	-0.27	0.72	1.72	0.090	0.191
Paraquat	4	4.44	3.16	-0.18	0.49	1.17	0.114	0.213
Monocrotaline	4	3.46	3.06	-0.37	0.18	0.73	0.368	0.499
Tenofovir	4	3.27	3.36	-0.80	-0.04	0.72	0.845	0.892
Carbamazepine	4	3.49	3.48	-0.34	0.00	0.34	0.989	0.989
Nitrofurantoin	16	20.87	4.14	1.71	2.33	2.95	0.001	0.006
Paraquat	16	47.29	4.46	2.48	3.41	4.33	0.001	0.006
Bicalutamide	16	5.69	4.34	-0.17	0.39	0.95	0.124	0.213
Nickel sulfate	16	4.03	4.72	-0.67	-0.23	0.22	0.206	0.326
Carbamazepine	16	5.14	4.34	-0.28	0.24	0.77	0.265	0.387
Diacetyl	16	4.66	4.22	-0.35	0.15	0.64	0.458	0.581
Tenofovir	16	4.79	4.34	-0.45	0.14	0.74	0.526	0.624
Monocrotaline	16	4.27	4.59	-0.57	-0.10	0.37	0.577	0.645

Table S5. Proposed lung adverse outcome pathways under development

AOP Wiki No.	AOP Title	OECD Status	Inflammation events?	Oxidative-stress events?
148	EGFR activation leading to decreased lung function	Under development	No	No
173	Increased substance interaction with the resident cell membrane components leading to lung fibrosis	Under development	Yes	No
196	Volatile organic chemicals activate TRPA1 receptor to induce sensory pulmonary irritation	NA	Yes	No
206	Peroxisome proliferator-activated receptors γ inactivation leading to lung fibrosis	Under development	Yes	No
241	Latent transforming growth factor beta1 activation leads to pulmonary fibrosis	NA	Unclear	No

Note: Based on information extracted from AOP Wiki (https://aopwiki.org/) on 3rd April, 2018

Reference

1. Mayne Pharma Inc. Amiodarone hydrochloride tablet [package insert] [Internet]. US NLM DailyMed; 2016 Oct. Available: https://dailymed.nlm.nih.gov/dailymed/drugInfo.cfm?setid=08641e51-abca-4c1c-ba19-d24435332018
2. Wilson BD, Jaworski AJ, Donner ME, Lippmann ML. Amiodarone-induced pulmonary toxicity in the rat. Lung. 1989;167: 301–311. doi:10.1007/BF02714959
3. Fresenius Kabi USA, LLC. Bleomycin sulfate injection, powder, lyophilized, for solution [package insert] [Internet]. US NLM DailyMed; 2017 Jan. Available: https://dailymed.nlm.nih.gov/dailymed/drugInfo.cfm?setid=b5806c40-12ce-48e3-8abd-9f8997ef4428
4. Moeller A, Ask K, Warburton D, Gauldie J, Kolb M. The bleomycin animal model: a useful tool to investigate treatment options for idiopathic pulmonary fibrosis? Int J Biochem Cell Biol. 2008;40: 362–382. doi:10.1016/j.biocel.2007.08.011
5. CARACO PHARMACEUTICAL LABORATORIES, LTD. Carbamazepine tablet [package insert] [Internet]. US NLM DailyMed; 2008 Jan. Available: https://dailymed.nlm.nih.gov/dailymed/drugInfo.cfm?setid=c13bc0b8-7900-4ef4-98ed-e1315a08d95d
6. Carbamazepine. In: US NLM Hazardous Substances Data Bank [Internet]. 13 Dec 2007 [cited 2 Jul 2017]. Available: https://toxnet.nlm.nih.gov/cgi-bin/sis/search2/r?dbs+hsdb:@term+@DOCNO+3019
7. Wilschut FA, Cobben NA, Thunnissen FB, Lamers RJ, Wouters EF, Drent M. Recurrent respiratory distress associated with carbamazepine overdose. Eur Respir J. 1997;10: 2163–2165.
8. Kitson GE, Wauchob TD. Pulmonary oedema following carbamazepine overdose. Anaesthesia. 1988;43: 967–969. doi:10.1111/j.1365-2044.1988.tb05665.x
9. Ciba-Geigy Corporation. FDA Pharmacologist Review of NDA 16-608 (Tegretol) [Internet]. 1967 Dec. Available: https://www.accessdata.fda.gov/drugsatfda_docs/nda/pre96/016608_Pharm_rvw2.pdf
10. Roxane Laboratories, Inc. Cyclophosphamide tablet [package insert] [Internet]. US NLM DailyMed; 2006 Nov. Available: https://dailymed.nlm.nih.gov/dailymed/drugInfo.cfm?setid=367b47d7-c4de-4b39-bd3a-69c29d80396f
11. Patel JM. Cyclophosphamide: pulmonary metabolism, toxicity and protective effect of vitamin e. In: Gram TE, editor. Metabolic Activation and Toxicity of Chemical Agents to Lung Tissue and Cells. Amsterdam: Pergamon; 1993. pp. 239–254. doi:10.1016/B978-0-08-041177-4.50018-3
12. Siemann DW, Macler L, Penney DP. Cyclophosphamide-induced pulmonary toxicity. Br J Cancer Suppl. 1986;7: 343–346.
13. Cyclophosphamide [Internet]. National Toxicology Program (NTP) Report on Carcinogens 14th Edition; 2016. Available: https://ntp.niehs.nih.gov/ntp/roc/content/profiles/cyclophosphamide.pdf
14. Fresenius Kabi USA, LLC. Methotrexate sodium injection, powder, lyophilized, for solution [package insert] [Internet]. US NLM DailyMed; 2016 Apr. Available: https://dailymed.nlm.nih.gov/dailymed/drugInfo.cfm?setid=ec476b11-0b3c-4139-b1eb-a3daa76bc271#a9b7e966-af5e-44a9-bb63-a00d00547551
15. Lateef O, Shakoor N, Balk RA. Methotrexate pulmonary toxicity. Expert Opin Drug Saf. 2005;4: 723–730. doi:10.1517/14740338.4.4.723
16. Ohbayashi M, Suzuki M, Yashiro Y, Fukuwaka S, Yasuda M, Kohyama N, et al. Induction of pulmonary fibrosis by methotrexate treatment in mice lung in vivo and in vitro. J Toxicol Sci. 2010;35: 653–661.
17. Methotrexate. In: US NLM Hazardous Substances Data Bank [Internet]. 14 Nov 2005 [cited 2 Jul 2017]. Available: https://toxnet.nlm.nih.gov/cgi-bin/sis/search2/r?dbs+hsdb:@term+@DOCNO+3123
18. Actavis Pharma, Inc. Nitrofurantoin suspension [package insert] [Internet]. US NLM DailyMed; 2016 Jan. Available: https://dailymed.nlm.nih.gov/dailymed/drugInfo.cfm?setid=d8c5b015-626e-4d57-9b91-392fb53575fa
19. Boyd MR, Catignani GL, Sasame HA, Mitchell JR, Stiko AW. Acute Pulmonary Injury in Rats by Nitrofurantoin and Modification by Vitamin E, Dietary Fat, and Oxygen. Am Rev Respir Dis. 1979;120: 93–99. doi:10.1164/arrd.1979.120.1.93
20. Toxicology and carcinogenesis studies of Nitrofurantoin (CAS No. 67-20-9) in F344/N rats and B6C3F1 mice (feed studies). National Toxicology Program (NTP) Technical Report No 341. 1989; Available: https://ntp.niehs.nih.gov/ntp/htdocs/lt_rpts/tr341.pdf
21. Wyeth Pharmaceuticals Inc. Temsirolimus [package insert] [Internet]. US NLM DailyMed; 2017 Jun. Available: https://dailymed.nlm.nih.gov/dailymed/drugInfo.cfm?setid=95b7dc92-2180-42f1-8699-3c28f609e674
22. Sun Y, Rha S, Lee S-H, Guo J, Ueda T, Qin S, et al. Phase II Study of the Safety and Efficacy of Temsirolimus in East Asian Patients with Advanced Renal Cell Carcinoma. Jpn J Clin Oncol. 2012;42: 836–844. doi:10.1093/jjco/hys110
23. Wyeh Pharmaceuticals, Inc. FDA Pharmacologist Review of NDA 22-088 (Temsirolimus) [Internet]. 2007 May. Available: https://www.accessdata.fda.gov/drugsatfda_docs/nda/pre96/016608_Pharm_rvw2.pdf

24. Torisel: EPAR - Scientific Discussion. In: European Medicines Agency Committee for Medicinal Products for Human Use (CHMP) initial marketing-authorisation documents [Internet]. 5 Dec 2007 [cited 2 Jul 2017]. Available: http://www.ema.europa.eu/docs/en_GB/document_library/EPAR_-_Scientific_Discussion/human/000799/WC500039915.pdf

25. State of Florida DOH Central Pharmacy. Tenofovir disoproxil fumarate tablet, coated [package insert] [Internet]. US NLM DailyMed; 2010 Apr. Available: https://dailymed.nlm.nih.gov/dailymed/drugInfo.cfm?setid=e122435e-cd0b-4c90-940a-b7a0d090d866

26. Gilead Sciences. FDA Briefing Document of NDA 21-356 (Tenofovir Disoproxil Fumarate) [Internet]. 2001 Aug. Available: https://www.fda.gov/ohrms/dockets/ac/01/briefing/3792b1_01_gilead.pdf

27. Bennett CL. Pneumonitis Associated with Nonsteroidal Antiandrogens: Presumptive Evidence of a Class Effect. Ann Intern Med. 2002;137: 625. doi:10.7326/0003-4819-137-7-200210010-00029

28. Rodriguez EM, Staffa JA, Graham DJ. The role of databases in drug postmarketing surveillance. Pharmacoepidemiol Drug Saf. 2001;10: 407–410. doi:10.1002/pds.615

29. Iswaran TJ, Imai M, Betton GR, Siddall RA. An Overview of Animal Toxicology Studies with Bicalutamide (ici 176, 334). J Toxicol Sci. 1997;22: 75–88. doi:10.2131/jts.22.2_75

30. Bicalutamide. In: US NLM Hazardous Substances Data Bank [Internet]. 5 May 2009 [cited 30 Jun 2017]. Available: http://toxnet.nlm.nih.gov/cgi-bin/sis/search2/r?dbs+hsdb:@term+@DOCNO+7655

31. Wilson R, Welte T, Polverino E, Soyza AD, Greville H, O'Donnell A, et al. Ciprofloxacin dry powder for inhalation in non-cystic fibrosis bronchiectasis: a phase II randomised study. Eur Respir J. 2013;41: 1107–1115. doi:10.1183/09031936.00071312

32. Testpak Holding Company. Ciprofloxacin hydrochloride tablet, coated [package insert] [Internet]. US NLM DailyMed; 2010 Feb. Available: https://dailymed.nlm.nih.gov/dailymed/drugInfo.cfm?setid=8c353bf4-f26b-4ab8-9694-aa9f10dfe965

33. Ciprofloxacin. In: US NLM Hazardous Substances Data Bank [Internet]. 12 Oct 2012 [cited 1 Jul 2017]. Available: http://toxnet.nlm.nih.gov/cgi-bin/sis/search2/r?dbs+hsdb:@term+@DOCNO+6987

34. Schluter G. Ciprofloxacin: toxicologic evaluation of additional safety data. Am J Med. 1989;87: 37S–39S.

35. Gould S, Scott RC. 2-Hydroxypropyl-β-cyclodextrin (HP-β-CD): A toxicology review. Food Chem Toxicol. 2005;43: 1451–1459. doi:10.1016/j.fct.2005.03.007

36. Ketoconazole HRA: EPAR - Public assessment report. In: European Medicines Agency Committee for Medicinal Products for Human Use (CHMP) initial marketing-authorisation documents [Internet]. 25 Sep 2014 [cited 1 Jul 2017]. Available: http://www.ema.europa.eu/docs/en_GB/document_library/EPAR_-_Public_assessment_report/human/003906/WC500181493.pdf

37. Sugar AM, Alsip SG, Galgiani JN, Graybill JR, Dismukes WE, Cloud GA, et al. Pharmacology and toxicity of high-dose ketoconazole. Antimicrob Agents Chemother. 1987;31: 1874–1878.

38. Mylan Pharmaceuticals Inc. Ketoconazole tablet [package insert] [Internet]. US NLM DailyMed; 2014 Apr. Available: https://dailymed.nlm.nih.gov/dailymed/drugInfo.cfm?setid=46698e84-8dd2-4faf-aaf0-d7cddfebb71f

39. X-GEN Pharmaceuticals, Inc. Lincomycin hydrochloride injection, solution [package insert] [Internet]. US NLM DailyMed; 2015 Oct. Available: https://dailymed.nlm.nih.gov/dailymed/drugInfo.cfm?setid=61f4851d-d919-400d-912a-05eb9dca11d4

40. Gray JE, Purmalis A, Feenstra ES. Animal toxicity studies of a new antibiotic, lincomycin. Toxicol Appl Pharmacol. 1964;6: 476–496. doi:10.1016/S0041-008X(64)80014-4

41. Melatonin. In: US NLM Hazardous Substances Data Bank [Internet]. 4 Sep 2014 [cited 2 Jul 2017]. Available: https://toxnet.nlm.nih.gov/cgi-bin/sis/search2/r?dbs+hsdb:@term+@DOCNO+7509

42. Apotheca Company. Melatonin, liquid [package insert] [Internet]. US NLM DailyMed; 2011 Jul. Available: https://dailymed.nlm.nih.gov/dailymed/drugInfo.cfm?setid=fdaf86c2-137d-4926-a63b-9faf7ec7e04c

43. Goyal A, Terry PD, Superak HM, Nell-Dybdahl CL, Chowdhury R, Phillips LS, et al. Melatonin supplementation to treat the metabolic syndrome: a randomized controlled trial. Diabetol Metab Syndr. 2014;6: 124. doi:10.1186/1758-5996-6-124

44. Accord Healthcare, Inc. Naltrexone hydrochloride tablet, film coated [package insert] [Internet]. US NLM DailyMed; 2017 Feb. Available: https://dailymed.nlm.nih.gov/dailymed/drugInfo.cfm?setid=49aa3d6d-2270-4615-aafa-b440859ab870

45. Narcotic antagonists: Naltrexone progress report. National Institute on Drug Abuse Research Monograph 9. 1976; Available: https://archives.drugabuse.gov/pdf/monographs/09.pdf

46. Alvogen Inc. Nevirapine tablet [package insert] [Internet]. US NLM DailyMed; 2013 May. Available: https://dailymed.nlm.nih.gov/dailymed/drugInfo.cfm?setid=5d5d8d1c-f418-455a-c693-c82a79df3637

47. Patel SM, Johnson S, Belknap SM, Chan J, Sha BE, Bennett C. Serious adverse cutaneous and hepatic toxicities associated with nevirapine use by non-HIV-infected individuals. J Acquir Immune Defic Syndr 1999. 2004;35: 120–125.

48. Heritage Pharmaceuticals Inc. Nystatin tablet, coated [package insert] [Internet]. US NLM DailyMed; 2016 Mar. Available: https://dailymed.nlm.nih.gov/dailymed/drugInfo.cfm?setid=e87b8480-987d-4f82-87bf-cf831d6984a1

49. Nystatin. In: US NLM Hazardous Substances Data Bank [Internet]. 20 Dec 2006 [cited 2 Jul 2017]. Available: https://toxnet.nlm.nih.gov/cgi-bin/sis/search2/r?dbs+hsdb:@term+@DOCNO+3138

50. Mehta RT, Hopfer RL, McQueen T, Juliano RL, Lopez-Berestein G. Toxicity and therapeutic effects in mice of liposome-encapsulated nystatin for systemic fungal infections. Antimicrob Agents Chemother. 1987;31: 1901–1903. doi:10.1128/AAC.31.12.1901

51. Drouhet E, Schwarz J, Bingham E. Evaluation of the action of nystatin on Histoplasma capsulatum in vitro and in hamsters and mice. Antibiot Chemother Northfield Ill. 1956;6: 23–35.

52. Phenacetin. In: US NLM Hazardous Substances Data Bank [Internet]. 11 Oct 2007 [cited 2 Jul 2017]. Available: https://toxnet.nlm.nih.gov/cgi-bin/sis/search2/r?dbs+hsdb:@term+@DOCNO+3152

53. Phenacetin and analgesic mixtures containing phenacetin [Internet]. National Toxicology Program (NTP) Report on Carcinogens 14th Edition; 2016. Available: https://ntp.niehs.nih.gov/ntp/roc/content/profiles/phenacetinandanalgesicmixtures.pdf

54. Xylazine. WHO Food Additives Series 38. 1996; Available: http://www.inchem.org/documents/jecfa/jecmono/v38je03.htm

55. Poklis A, Mackell MA, Case MES. Xylazine in Human Tissues and Fluids in a Case of Fatal Drug Abuse. J Anal Toxicol. 1985;9: 234–236. doi:10.1093/jat/9.5.234

56. Amouzadeh HR, Sangiah S, Qualls CW, Cowell RL, Mauromoustakos A. Xylazine-induced pulmonary edema in rats. Toxicol Appl Pharmacol. 1991;108: 417–427. doi:10.1016/0041-008X(91)90088-V

57. Final report on carcinogens background document for aristolochic acids [Internet]. National Toxicology Program (NTP); 2008 Sep. Available: https://ntp.niehs.nih.gov/ntp/roc/twelfth/2010/finalbds/aristolochic_acids_final_508.pdf

58. Arlt VM, Alunni-Perret V, Quatrehomme G, Ohayon P, Albano L, Gaïd H, et al. Aristolochic acid (AA)-DNA adduct as marker of AA exposure and risk factor for AA nephropathy-associated cancer. Int J Cancer. 2004;111: 977–980. doi:10.1002/ijc.20316

59. Kreiss K, Gomaa A, Kullman G, Fedan K, Simoes EJ, Enright PL. Clinical Bronchiolitis Obliterans in Workers at a Microwave-Popcorn Plant. N Engl J Med. 2002;347: 330–338. doi:10.1056/NEJMoa020300

60. van Rooy FGBGJ, Rooyackers JM, Prokop M, Houba R, Smit LAM, Heederik DJJ. Bronchiolitis Obliterans Syndrome in Chemical Workers Producing Diacetyl for Food Flavorings. Am J Respir Crit Care Med. 2007;176: 498–504. doi:10.1164/rccm.200611-1620OC

61. Morgan DL, Flake GP, Kirby PJ, Palmer SM. Respiratory Toxicity of Diacetyl in C57Bl/6 Mice. Toxicol Sci. 2008;103: 169–180. doi:10.1093/toxsci/kfn016

62. Hubbs AF, Goldsmith WT, Kashon ML, Frazer D, Mercer RR, Battelli LA, et al. Respiratory Toxicologic Pathology of Inhaled Diacetyl in Sprague-Dawley Rats. Toxicol Pathol. 2008;36: 330–344. doi:10.1177/0192623307312694

63. Grogan MW. Toxicity from BHT ingestion. West J Med. 1986;145: 245–246.

64. Shlian DM, Goldstone J. More on BHT toxicity. West J Med. 1986;145: 699.

65. Witschi H, Malkinson AM, Thompson JA. Metabolism and pulmonary toxicity of butylated hydroxytoluene. In: Gram TE, editor. Metabolic Activation and Toxicity of Chemical Agents to Lung Tissue and Cells. Amsterdam: Pergamon; 1993. pp. 185–212. doi:10.1016/B978-0-08-041177-4.50016-X

66. Sorbitol. WHO Food Additives Series 5. 1974; Available: http://www.inchem.org/documents/jecfa/jecmono/v05je91.htm

67. Pyrrolizidine alkaloids. WHO Environmental Health Criteria 80. 1988; Available: http://www.inchem.org/documents/ehc/ehc/ehc080.htm

68. Wiedenfeld H, Edgar J. Toxicity of pyrrolizidine alkaloids to humans and ruminants. Phytochem Rev. 2011;10: 137–151. doi:10.1007/s11101-010-9174-0

69. Wilson DW, Segall HJ, Pan LC, Lamé MW, Estep JE, Morin D. Mechanisms and Pathology of Monocrotaline Pulmonary Toxicity. Crit Rev Toxicol. 1992;22: 307–325. doi:10.3109/10408449209146311

70. Huxtable RJ. Hepatic nonaltruism and pulmonary toxicity of pyrrolizidine alkaloids. In: Gram TE, editor. Metabolic Activation and Toxicity of Chemical Agents to Lung Tissue and Cells. Amsterdam: Pergamon; 1993. pp. 213–237. doi:10.1016/B978-0-08-041177-4.50017-1

71. Sodium chloride. In: US NLM Hazardous Substances Data Bank [Internet]. 4 Sep 2014 [cited 2 Jul 2017]. Available: https://toxnet.nlm.nih.gov/cgi-bin/sis/search2/r?dbs+hsdb:@term+@DOCNO+6368

72. Pfohl-Leszkowicz A, Manderville RA. Ochratoxin A: An overview on toxicity and carcinogenicity in animals and humans. Mol Nutr Food Res. 2007;51: 61–99. doi:10.1002/mnfr.200600137

73. The endemic nephropathy of south-eastern Europe. Bull World Health Organ. 1965;32: 431–448.

74. Toxicology and carcinogenesis studies of Ochratoxin A (CAS No. 303-47-9) in F344/N rats (gavage studies). National Toxicology Program (NTP) Technical Report Series No 358. 1989; Available: https://ntp.niehs.nih.gov/ntp/htdocs/lt_rpts/tr358.pdf

75. Patulin. In: US NLM Hazardous Substances Data Bank [Internet]. 18 Dec 2009 [cited 30 Jun 2017]. Available: http://toxnet.nlm.nih.gov/cgi-bin/sis/search2/r?dbs+hsdb:@term+@DOCNO+3522

76. Hayes AW, Phillips TD, Williams WL, Ciegler A. Acute toxicity of patulin in mice and rats. Toxicology. 1979;13: 91–100. doi:10.1016/S0300-483X(79)80014-1

77. Reddy CS, Chan PK, Hayes AW, Williams WL, Ciegler A. Acute toxicity of patulin and its interaction with penicillic acid in dogs. Food Cosmet Toxicol. 1979;17: 605–609. doi:10.1016/0015-6264(79)90120-2

78. Toxicological profile for Cadmium [Internet]. U.S. Department of Health and Human Services, Agency for Toxic Substances and Disease Registry (ATSDR); 2012 Sep. Available: https://www.atsdr.cdc.gov/toxprofiles/tp5.pdf

79. Toxicological profile for Nickel [Internet]. U.S. Department of Health and Human Services, Agency for Toxic Substances and Disease Registry (ATSDR); 2005 Aug. Available: https://www.atsdr.cdc.gov/toxprofiles/tp15.pdf

80. Toxicology and carcinogenesis studies of Nickel Sulfate Hexahydrate (CAS No. 10101-97-0) in F344/N rats and B6C3F1 mice (inhalation studies). National Toxicology Program (NTP) Technical Report No 454. 1996; Available: https://ntp.niehs.nih.gov/ntp/htdocs/lt_rpts/tr454.pdf

81. Toxicological profile for Ethylene Glycol [Internet]. U.S. Department of Health and Human Services, Agency for Toxic Substances and Disease Registry (ATSDR); 2010 Nov. Available: https://www.atsdr.cdc.gov/toxprofiles/tp96.pdf

82. Lubner SJ, Kunnimalaiyaan M, Holen KD, Ning L, Ndiaye M, LoConte NK, et al. A Preclinical and Clinical Study of Lithium in Low-Grade Neuroendocrine Tumors. The Oncologist. 2011;16: 452–457. doi:10.1634/theoncologist.2010-0323

83. Radomski JL, Fuyat HN, Nelson AA, Smith PK. The Toxic Effects, Excretion and Distribution of Lithium Chloride. J Pharmacol Exp Ther. 1950;100: 429–444.

84. Lithium chloride. In: US NLM Hazardous Substances Data Bank [Internet]. 11 Oct 2007 [cited 1 Jul 2017]. Available: https://toxnet.nlm.nih.gov/cgi-bin/sis/search2/r?dbs+hsdb:@term+@DOCNO+4281

85. Toxicological profile for 1,1-Dichloroethene [Internet]. U.S. Department of Health and Human Services, Agency for Toxic Substances and Disease Registry (ATSDR); 1994 May. Available: https://www.atsdr.cdc.gov/toxprofiles/tp39.pdf

86. 1,1-Dichloroethylene. In: US NLM Hazardous Substances Data Bank [Internet]. 26 Jun 2009 [cited 30 Jun 2017]. Available: http://toxnet.nlm.nih.gov/cgi-bin/sis/search2/r?dbs+hsdb:@term+@DOCNO+1995

87. Final Report on the Safety Assessment of Triethylene Glycol and PEG-41. Int J Toxicol. 2006;25: 121–138. doi:10.1080/10915810600964642

88. Butane-1,3-Diol. WHO Food Additives Series 14. 1980; Available: http://www.inchem.org/documents/jecfa/jecmono/v14je03.htm

89. Dipropylene Glycol. In: US NLM Hazardous Substances Data Bank [Internet]. 7 Apr 2015 [cited 1 Jul 2017]. Available: https://toxnet.nlm.nih.gov/cgi-bin/sis/search2/r?dbs+hsdb:@term+@DOCNO+2658

90. Fowles JR, Banton MI, Pottenger LH. A toxicological review of the Propylene Glycols. Crit Rev Toxicol. 2013;43: 363–390. doi:10.3109/10408444.2013.792328

91. Addendum for Propylene Glycol supplement to the 1997 toxicological profile for Propylene Glycol [Internet]. U.S. Department of Health and Human Services, Agency for Toxic Substances and Disease Registry (ATSDR); 2008 Dec. Available: https://www.atsdr.cdc.gov/toxprofiles/propylene_glycol_addendum.pdf

92. Toxicology and carcinogenesis studies of Methyl trans-styryl Ketone (CAS No. 1896-62-4) in F344/N rats and B6C3F1 mice (feed and dermal studies). National Toxicology Program (NTP) Technical Report Series No 572. 2012; Available: https://ntp.niehs.nih.gov/ntp/htdocs/lt_rpts/tr572_508.pdf

93. Dibutyl phthalate. In: US NLM Hazardous Substances Data Bank [Internet]. 19 Oct 2015 [cited 30 Jun 2017]. Available: http://toxnet.nlm.nih.gov/cgi-bin/sis/search2/r?dbs+hsdb:@term+@DOCNO+922

94. Toxicity studies of Dibutyl Phthalate (CAS No. 84-74-2) administered in feed to F344/N rats and B6C3F1 mice. National Toxicology Program (NTP) Toxicity Report Series No 30. 1995; Available: https://ntp.niehs.nih.gov/ntp/htdocs/st_rpts/tox030.pdf

95. Walseth F, Toftgård R, Nilsen OG. Phthalate esters I: Effects on cytochrome P-450 mediated metabolism in rat liver and lung, serum enzymatic activities and serum protein levels. Arch Toxicol. 1982;50: 1–10. doi:10.1007/BF00569231

96. Walseth F, Nilsen OG. Phthalate esters II: Effects of inhaled dibutylphthalate on cytochrome P-450 mediated metabolism in rat liver and lung. Arch Toxicol. 1984;55: 132–136. doi:10.1007/BF00346052

97. Toxicological profile for Diethyl Phthalate [Internet]. U.S. Department of Health and Human Services, Agency for Toxic Substances and Disease Registry (ATSDR); 1995 Jun. Available: https://www.atsdr.cdc.gov/toxprofiles/tp73.pdf

98. Diethyl phthalate. WHO International Programme on Chemical Safety, Concise international chemical assessment document 52. 2003; Available: http://www.who.int/ipcs/publications/cicad/en/cicad52.pdf

99. 3-Methylindole. In: US NLM Hazardous Substances Data Bank [Internet]. 14 Feb 2003 [cited 30 Jun 2017]. Available: http://toxnet.nlm.nih.gov/cgi-bin/sis/search2/r?dbs+hsdb:@term+@DOCNO+3511

100. Bray TM, Emmerson KS. Putative Mechanisms of Toxicity of 3-Methylindole: From Free Radical to Pneumotoxicosis. Annu Rev Pharmacol Toxicol. 1994;34: 91–115. doi:10.1146/annurev.pa.34.040194.000515

101. Bray TM, Kirkland JB. The metabolic basis of 3-Methylindole-induced pneumotoxicity. In: Gram TE, editor. Metabolic Activation and Toxicity of Chemical Agents to Lung Tissue and Cells. Amsterdam: Pergamon; 1993. pp. 165–184. doi:10.1016/B978-0-08-041177-4.50015-8

102. Yost GS. Mechanisms of 3-methylindole pneumotoxicity. Chem Res Toxicol. 1989;2: 273–279. doi:10.1021/tx00011a001

103. Final Amended Report on the Safety Assessment of Methylparaben, Ethylparaben, Propylparaben, Isopropylparaben, Butylparaben, Isobutylparaben, and Benzylparaben as used in Cosmetic Products. Int J Toxicol. 2008;27: 1–82. doi:10.1177/109158180802704s01

104. Myrcene. In: US NLM Hazardous Substances Data Bank [Internet]. 26 Apr 2012 [cited 30 Jun 2017]. Available: http://toxnet.nlm.nih.gov/cgi-bin/sis/search2/r?dbs+hsdb:@term+@DOCNO+1258

105. Toxicology and carcinogenesis studies of β-Myrcene (CAS No. 123-35-3) in F344/N rats and B6C3F1 mice (gavage studies). National Toxicology Program (NTP) Technical Report No 557. 2010; Available: https://ntp.niehs.nih.gov/ntp/htdocs/lt_rpts/tr557.pdf

106. Abidi K, Himdi B, Cherradi N, Lamalmi N, Alhamany Z, Zeggwagh A, et al. Myocardial lysis in a fetus induced by maternal paraphenylenediamine poisoning following an intentional ingestion to induce abortion. Hum Exp Toxicol. 2008;27: 435–438. doi:10.1177/0960327108092288

107. Behera C, Mridha AR, Kumar R, Millo T. Characteristic autopsy findings in hair dye poisoning. BMJ Case Rep. 2015;2015: bcr2014206692. doi:10.1136/bcr-2014-206692

108. Elgamel A, Ahmed N. Complications and management of hair dye poisoning in Khartoum. Sudan Med Monit. 2013;8: 146. doi:10.4103/1858-5000.132603

109. Opinion on p-Phenylenediamine. EC Scientific Committee on Consumer Products. 2006;SCCP/0989/06. Available: https://ec.europa.eu/health/ph_risk/committees/04_sccp/docs/sccp_o_069.pdf

110. Safety assessment of p-Phenylenediamine, p-Phenylenediamine HCl, and p-Phenylenediamine Sulfate. Amend Final Rep Cosmet Ingred Rev Expert Panel. 2007; Available: http://gov.personalcarecouncil.org/ctfa-static/online/lists/cir-pdfs/FR530.pdf

111. Katsumi I, Yuichi I, Osamu N, Keisuke N, Nobuyuki I. Carcinogenicity and toxicity tests on p-phenylenediamine in F344 rats. Toxicol Lett. 1983;16: 259–269. doi:10.1016/0378-4274(83)90186-8

112. Maronpot RR, Shimkin MB, Witschi HP, Smith LH, Cline JM. Strain A Mouse Pulmonary Tumor Test Results for Chemicals Previously Tested in the National Cancer Institute Carcinogenicity Tests. JNCI J Natl Cancer Inst. 1986;76: 1101–1112. doi:10.1093/jnci/76.6.1101

113. Smith P, Heath D. Paraquat lung: a reappraisal. Thorax. 1974;29: 643–653. doi:10.1136/thx.29.6.643

114. Dinis-Oliveira RJ, Duarte JA, Sánchez-Navarro A, Remião F, Bastos ML, Carvalho F. Paraquat Poisonings: Mechanisms of Lung Toxicity, Clinical Features, and Treatment. Crit Rev Toxicol. 2008;38: 13–71. doi:10.1080/10408440701669959

115. Murray RE, Gibson JE. A comparative study of paraquat intoxication in rats, guinea pigs and monkeys. Exp Mol Pathol. 1972;17: 317–325. doi:10.1016/0014-4800(72)90044-5

116. Toxicological profile for DDT, DDE, and DDD [Internet]. U.S. Department of Health and Human Services, Agency for Toxic Substances and Disease Registry (ATSDR); 2002 Sep. Available: https://www.atsdr.cdc.gov/toxprofiles/tp35.pdf

117. Cocco P, Fadda D, Billai B, D'Atri M, Melis M, Blair A. Cancer Mortality among Men Occupationally Exposed to Dichlorodiphenyltrichloroethane. Cancer Res. 2005;65: 9588–9594. doi:10.1158/0008-5472.CAN-05-1487

118. DDT. INCHEM Poisons Information Monograph 127. 1990; Available: http://www.inchem.org/documents/pims/chemical/pim127.htm

119. Rose JE, Behm FM, Drgon T, Johnson C, Uhl GR. Personalized Smoking Cessation: Interactions between Nicotine Dose, Dependence and Quit-Success Genotype Score. Mol Med. 2010;16: 247–253. doi:10.2119/molmed.2009.00159

120. Ebbert JO, Croghan IT, Schroeder DR, Hurt RD. A Randomized Phase II Clinical Trial of High-Dose Nicotine Patch Therapy for Smokeless Tobacco Users. Nicotine Tob Res. 2013;15: 2037–2044. doi:10.1093/ntr/ntt097

121. Solarino B, Rosenbaum F, Rießelmann B, Buschmann CT, Tsokos M. Death due to ingestion of nicotine-containing solution: Case report and review of the literature. Forensic Sci Int. 2010;195: e19–e22. doi:10.1016/j.forsciint.2009.11.003

122. Waldum HL, Nilsen OG, Nilsen T, Rørvik H, Syversen U, Sandvik AK, et al. Long-term effects of inhaled nicotine. Life Sci. 1996;58: 1339–1346. doi:10.1016/0024-3205(96)00100-2

123. 1-Nitronaphthalene. In: US NLM Hazardous Substances Data Bank [Internet]. 2 Mar 2004 [cited 30 Jun 2017]. Available: http://toxnet.nlm.nih.gov/cgi-bin/sis/search2/r?dbs+hsdb:@term+@DOCNO+2887

124. Johnson DE, Riley MGI, Cornish HH. Acute target organ toxicity of 1-nitronaphthalene in the rat. J Appl Toxicol. 1984;4: 253–257. doi:10.1002/jat.2550040508

125. Sauer J-M, Eversole RR, Lehmann CL, Johnson DE, Beuving LJ. An ultrastructural evaluation of acute 1-nitronaphthalene induced hepatic and pulmonary toxicity in the rat. Toxicol Lett. 1997;90: 19–27. doi:10.1016/S0378-4274(96)03817-9

126. Toxicological profile for Naphthalene, 1-Methylnaphthalene, and 2-Methylnaphthalene [Internet]. U.S. Department of Health and Human Services, Agency for Toxic Substances and Disease Registry (ATSDR); 2015 Aug. Available: https://www.atsdr.cdc.gov/toxprofiles/tp67.pdf

127. Naphthalene [Internet]. National Toxicology Program (NTP) Report on Carcinogens 14th Edition; 2016. Available: https://ntp.niehs.nih.gov/ntp/roc/content/profiles/naphthalene.pdf

128. H C Lim, V Poulose, H H Tan. Acute naphthalene poisoning following the non-accidental ingestion of mothballs. Singapore Med J. 2009;50: 298.

129. N-Nitrosamines: 15 listings [Internet]. National Toxicology Program (NTP) Report on Carcinogens 14th Edition; 2016. Available: https://ntp.niehs.nih.gov/ntp/roc/content/profiles/nitrosamines.pdf

130. 4-(N-Nitrosomethylamino)-1-(3-pyridyl)-1-butanone. In: US NLM Hazardous Substances Data Bank [Internet]. 3 Jun 2010 [cited 30 Jun 2017]. Available: http://toxnet.nlm.nih.gov/cgi-bin/sis/search2/r?dbs+hsdb:@term+@DOCNO+7771

131. Pesticide residues in food - 2010: toxicological evaluation. Joint Meeting of the FAO Panel of Experts on Pesticide Residues in Food and the Environment and the WHO Core Assessment Group on Pesticide Residues. 2010;
